# Enrichments/Derichments of Root-Associated Bacteria Related to Plant Growth and Nutrition Caused by the Growth of an *EPSPS*-Transgenic Maize Line in the Field

**DOI:** 10.3389/fmicb.2019.01335

**Published:** 2019-06-18

**Authors:** Zhong-Ling Wen, Min-Kai Yang, Mei-Hang Du, Zhao-Zhao Zhong, Yun-Ting Lu, Gu-Hao Wang, Xiao-Mei Hua, Aliya Fazal, Chun-Hua Mu, Shu-Feng Yan, Yan Zhen, Rong-Wu Yang, Jin-Liang Qi, Zhi Hong, Gui-Hua Lu, Yong-Hua Yang

**Affiliations:** ^1^State Key Laboratory of Pharmaceutical Biotechnology, School of Life Sciences, Institute for Plant Molecular Biology, Nanjing University, Nanjing, China; ^2^Co-Innovation Center for Sustainable Forestry in Southern China, Nanjing Forestry University, Nanjing, China; ^3^Research Center for Soil Pollution Prevention and Control, Nanjing Institute of Environmental Sciences, MEE, Nanjing, China; ^4^Shandong Academy of Agriculture Sciences, Jinan, China; ^5^Henan Academy of Agriculture Sciences, Zhengzhou, China

**Keywords:** enrichments, derichments, root-associated bacteria, *EPSPS*-transgenic maize, nitrogen/phosphorus cycling

## Abstract

During the past decades, the effects of the transgenic crops on soil microbial communities have aroused widespread interest of scientists, which was mainly related to the health and growth of plants. In this study, the maize root-associated bacterial communities of *5-enolpyruvylshikimate-3-phosphate synthase* (*EPSPS*) transgenic glyphosate-tolerant (GT) maize line CC-2 (CC2) and its recipient variety Zhengdan958 (Z958) were compared at the tasseling and flowering stages by high-throughput sequencing of V3-V4 hypervariable regions of 16S rRNA gene (16S rDNA) amplicons via Illumina MiSeq. In addition, real-time quantitative PCR (qPCR) was also performed to analyze the *nifH* gene abundance between CC2 and Z958. Our results showed no significant difference in alpha/beta diversity of root-associated bacterial communities at the tasseling or flowering stage between CC2 and Z958 under field growth conditions. The relative abundances of the genera *Bradyrhizobium* and *Bacillus* including species *B. cereus* and *B. muralis* were significantly lower in the roots of CC2 than that of Z985 under field conditions. Both these species are regarded as plant growth promoting bacteria (PGPB), as they belong to both nitrogen-fixing and phosphate-solubilizing bacterial genera. The comparison of the relative abundance of nitrogen-fixing/phosphate-solubilizing bacteria at the class, order or family levels indicated that only one class Bacilli, one order Bacillales and one family *Bacillaceae* were found to be significantly lower in the roots of CC2 than that of Z985. These bacteria were also enriched in the roots and rhizospheric soil than in the surrounding soil at both two stages. Furthermore, the class Betaproteobacteria, the order Burkholderiales, the family *Comamonadaceae*, and the genus *Acidovorax* were significantly higher in the roots of CC2 than that of Z985 at the tasseling stage, meanwhile the order Burkholderiales and the family *Comamonadaceae* were also enriched in the roots than in the rhizospheric soil at both stages. Additionally, the *nifH* gene abundance at the tasseling stage in the rhizosphere soil also showed significant difference. The relative abundance of *nifH* gene was higher in the root samples and lower in the surrounding soil, which implicated that the roots of maize tend to be enriched in nitrogen-fixing bacteria.

## Introduction

The global commercial cultivation of genetically modified (GM) or transgenic crops has increased about 110-folds, from 1.7 million hectares (1996) to 185.1 million hectares (2016), and has already accumulated 2.1 billion hectares or 5.3 billion acres in 21 years (ISAAA, 2017). According to The International Service for the Acquisition of Agri-biotech Applications, herbicide tolerance deployed in soybean, maize, etc., has consistently been the dominant trait at 47% of the global hectarage (ISAAA, 2017). Although, these commercial transgenic crop varieties benefited economic gains of 574 million tons valued at US$167.8 billion in 1996-2015 and provided accessible food and nutrition to the 7.4 billion global populations (ISAAA, 2017), large amounts of previous studies also documented various effects of these transgenic crops on soil microbial communities especially rhizosphere bacteria (Dunfield and Germida, 2004; Liu et al., 2005; Turrini et al., 2015; Guan et al., 2016).

Rhizosphere microorganisms, such as bacteria and fungi, play important roles in improving plant health and growth (Lugtenberg and Kamilova, 2009; Berendsen et al., 2012; Bulgarelli et al., 2013; Berg et al., 2014), and in turn plants can strongly influence the composition, structure, and activity of rhizosphere microbiota, especially with respect to the active populations (Bais et al., 2006; Paterson et al., 2007; Aira et al., 2010; Bulgarelli et al., 2013; Kondorosi et al., 2013; Ofek et al., 2014). Moreover, the rhizosphere bacteria exhibit significant heritable variations between replicates of maize inbred lines (Peiffer et al., 2013). A recently study showed that rice genotypes not only affect the rhizosphere microbial communities, but also impact the microbial communities in the rhizoplane and endosphere (Edwards et al., 2015).

Previous studies reported that the release of numerous transgenic plants exert no or minor effects on soil microbial communities (Dunfield and Germida, 2004; Liu et al., 2005; Souza et al., 2013; Liang et al., 2014; Nakatani et al., 2014; Sohn et al., 2016). However, there are others that revealed significant effects of some GM plants on soil microbial communities. For example, the transgenic GT *Brassica napus* variety can significantly affect root-endophytic bacterial communities and rhizosphere microbial communities (Dunfield and Germida, 2001, 2003), the herbicide-tolerant transgenic Zoysia grass can affect the composition of rhizosphere microbial communities (Lee et al., 2011). The soil microbial taxonomic and functional abundances and the phylogenetic diversity of rhizosphere microbial communities were also affected by some glyphosate-resistant transgenic soybean lines (Babujia et al., 2016; Lu et al., 2017). Till date, numerous studies have been reported regarding the effects of transgenic plants on soil microbial communities, however further work needs to be conducted in this field as there is a scarcity of knowledge about the complex interactions between soil microbial communities and plants (Liu et al., 2005; Venturi and Keel, 2016). In addition, soil ecosystem is one of the complex and richest microbial communities on the earth (Gans et al., 2005; Tringe et al., 2005) so, an in-depth research is necessary to further evaluate the effects of transgenic plants on root-associated microbial communities.

To date, high-throughput next-generation sequencing (NGS) technologies, such as 454 GS FLX pyrosequencing, with its corresponding bioinformatics tools, are widely used in the research of soil microbial communities (Inceoglu et al., 2011; Bulgarelli et al., 2012, 2013; Lundberg et al., 2012; Peiffer et al., 2013; Schlaeppi et al., 2014). Meanwhile, the Illumina MiSeq platform has emerged as another powerful NGS technology for its far more cost-effective advantage and has been performed by many researchers for studying the highly complicated microbial communities (Kozich et al., 2013; Schmidt et al., 2013; Bakker et al., 2015; Edwards et al., 2015; Yang et al., 2015; Lu et al., 2018).

In this study, the root-associated bacterial communities of CC2 vs. Z958 were compared at tasseling and flowering stages under field growth conditions. For non-leguminous plants like maize and rice, the associative nitrogen-fixing bacteria play a more important role than symbiotic ones in the rhizospheric microorganisms related to nitrogen cycling. These main associative nitrogen-fixing bacterial genera in the rice roots were also found to be located in both rhizoplane and endosphere (Edwards et al., 2015). Thus, we further focused to compare the relative abundance of nitrogen-fixing and phosphate-solubilizing bacteria at molecular level in the maize root-associated bacterial communities, using 16S rDNA-based Illumina MiSeq high-throughput NGS platform and quantitative real-time PCR (qPCR) to deeply demonstrate the effects of transgenic maize on root-associated bacterial communities.

## Materials and Methods

### Plant Materials and Sampling Methods

The recipient maize cultivar was Zhengdan958 (Z958, Hybrid of Zheng58 × Chang7-2) and the *EPSPS* transgenic herbicide-tolerant maize line was CC2 (produced by the insertion of the maroACC gene) (Yan et al., 2018; Sun et al., 2019). The experimental fields were located in Yuanyang, Xinxiang City, Henan Province, China (N 35.011°, E 113.711°) and Zhangqiu, Jinan City, Shandong Province, China (N 36.780°, E 117.384°). The experimental samples were collected on August 9, 2016 from Yuanyang experimental field at the tasseling stage and on August 5, 2016 from Zhangqiu experimental field at the flowering stage. Before planting plants, the soil is sealed by spraying glyphosate, and then the weeding will not carried out before sampling. In both Yuanyang and Zhangqiu (tasseling and flowering stage), each maize cultivar was collected from four plots (5 × 4 m per plot) as four replicates randomly distributed over the field. In each plots, four plants were collected in the center according to the diagonal intersection as samples. Therefore, this means that the total number of plants collected are 32 (16 transgenic and 16 control) in one period. The plants were carefully excavated from the soil using a drain spade. Any roots that were at the interface of the plot and the soil were avoided in order to avoid false environments (Peiffer et al., 2013). After excavation, plants were placed in a plastic bag with several pre-freezing chemical ice packs and then immediately taken these samples to the laboratory. The sampling steps were done using the method previously described by Inceoglu and Lu with modifications (Inceoglu et al., 2010; Lu et al., 2018). Soil loosely adhering to the roots was shaken off from maize plant as surrounding soil samples and then stored at 4°C for enzyme activity analysis or at −80°C in a freezer for DNA extraction. Rhizosphere soil samples were collected by brushing off the soil tightly adhering to the root surface, then the root samples (samples of rhizoplane/endosphere, layer of endophytes in roots) were collected after being washed with phosphate buffered saline (PBS). Both of those samples were stored at −80°C in a freezer for DNA extraction.

### DNA Extraction From Soil and Root Samples

In this study, approximately 0.30 g of soil of every biological replicate were used to extracted total metagenomic DNA by using the PowerSoil DNA Isolation Kit (MoBio Laboratories Inc., Carlsbad, CA, USA), following the instructions with minor modifications, which means that the soil or root samples were homogenized in lysis buffer by using an LSE vortex mixer (LSE vortex mixer 230V; Corning Inc., USA) at 2,850 rpm for 10 min (Lu et al., 2017). Besides, approximately 2 × 0.7 g of maize root segments were carefully homogenized using a mortar and pestle under liquid nitrogen and the metagenomic DNA was extracted from homogenized root samples using the method above (Lu et al., 2017, 2018). After extraction, the quality of DNA samples was assessed on 1% agarose gel, and then subsequently quantified using a Qubit Fluorometer (Qubit 2.0, Invitrogen, Carlsbad, USA) to minimize the variability in surveys of microbial communities (Kennedy et al., 2014).

### 16S rDNA Amplicon Sequencing via Illumina MiSeq Platform

We used an improved dual-index high-throughput sequencing with paired-end 300nt and amplicons of approximately 469 bp encompassing the V3 and V4 hypervariable regions of the 16S rDNA (Fadrosh et al., 2014). The V3–V4 region of bacterial 16S rDNA was amplified using the following primers: forward primer 341F (5′-ACTCCTACGGGAGGCAGCAG-3′) and reverse primer 806R (5′-GGACTACHVGGGTWTCTAAT-3′) (Peiffer et al., 2013; Peng et al., 2014). Amplifying the V3-V4 regions has the following advantages: The primer sequence was designed with high coverage, number of known species in the target area is more, and the accuracy rate of species determination in the target area is high (Kozich et al., 2013; Sun et al., 2013; Fadrosh et al., 2014). The concentration of each qualified metagenomic DNA was more than 0.4 ng/μl, because the template concentration had a significant effect on the sample profile variability (Kennedy et al., 2014). PCR amplification, product purification, library quality determination and quantification were performed as previously described by Lu et al. (2017, 2018). The samples were sending to BGI Tech Solutions Co., Ltd. (Wuhan, China) to performed high-throughput sequencing of the qualified libraries on the Illumina MiSeq platform (Illumina, CA, USA) with MiSeq Reagent Kit. Total 48 sequencing clean data have already been submitted to the Sequence Read Archive (SRA) and the SRA accession number is PRJNA503294.

### Analysis of 16S rDNA Amplicon Sequencing Data

High-throughput sequencing of 16S rDNA (V3-V4 region) amplicons on the Illumina MiSeq platform was performed to characterize the bacterial community composition and structure in the surrounding soil, rhizospheric soil, and root samples of CC2 and Z958 at the tasseling and flowering stages. A total of 3,804,059 qualified paired-end clean reads with an average count per sample of 79,251 (range: 76,305–84,466) were obtained from surrounding soil, rhizospheric soil, and root samples at the tasseling and flowering stages (Supplementary Table 1). Among them, the total qualified paired clean reads at the tasseling stages were 1,896,033 and the average count per sample was 79,001 (range: 76,305–83,345). At flowering stage, the total qualified paired-end clean reads were 1,908,026 and the average count of per sample was 79,501 (range: 77,037–84,466) (Supplementary Table 1). The high quality paired-end reads were connected to tags based on overlaps, 3,674,677 tags were obtained in total with 76,555 tags per sample on average, and the average length was 458 bp. After the primer sequences were removed, tags without primers were 3,613,717 in total with 75,285 tags per sample on an average, and the average length was 418 bp (Supplementary Table 2). Operational Taxonomic Unit (OTU) was selected as previously described by Lu *et al*. with minor modifications (Lu et al., 2017). The clean tags were clustered into OTUs with a 97% similarity using software UPARSE (v7.0.1090) (Edgar, 2013), and the OTU unique representative sequences were obtained, then the chimeras were filtered out using UCHIME(v4.2.40) and 16S rDNA was screened for chimeras by mapping to gold database (v20110519) (Edgar et al., 2011). Then OTUs were filtered by removing OTUs which were unassigned or not assigned to the targeted species. The detailed information of OTUs was summarized in Supplementary Table 1 and OTU number per sample primarily represents the degree of sample diversity. As described by Lu et al. (2017), we normalized the OTU counts in each sample's library after species annotation and phylogenetic relation construction. Detailed information was summarized in Supplementary Table 3, OTU number per sample primarily represents the degree of sample diversity. The numbers of filtered tags were 2,068,305 in total with 43,089 tags per sample on average. In addition, 131,935 OTUs were identified with an average of 2,748 OTUs per sample (Supplementary Table 3). Then, alpha and beta diversity analyses were subsequently analyzed based on OTUs and species annotation results.

### Alpha Diversity, Beta Diversity, and Functional Analysis

Alpha diversity was applied for analyzing complexity of species diversity. It can be expressed by different indices, including observed species, chao1, ACE, shannon and simpson (Schloss et al., 2009). The complexity of sample is positively correlated with the first four indices while negatively correlated with simpson value. Beta diversity analysis was used to evaluate differences in species complexity between CC2 and Z958 through several values, such as Bray-Curtis, weighted-UniFrac and unweighted-UniFrac. Beta diversity was calculated using QIIME (v 1.8.0) (Caporaso et al., 2010) and beta diversity analysis was used to evaluate differences of samples in species complexity between CC2 and Z958. PCA (principal component analysis) is a technique for analyzing and simplifying data sets. The difference and distance between samples can be reflected by analyzing the composition of OTUs (97% similarity) of different samples. PCoA based on the weighted-UniFrac (WUF) and the Bray–Curtis distance was also performed with QIIME (v1.8.0). Unweighted Pair Group Method with Arithmetic mean (UPGMA) was conducted with QIIME (v 1.8.0) using average linkage as a type of hierarchical clustering method and was used to interpret the distance matrix produced by beta diversity and the figure was drawn by software R (v3.1.3). Beta diversity heat map was drawn by “a heat map” in package “NMF” software R (v3.1.3). Taxa clustering is based on the relative abundance of each taxon; where the longitudinal clustering indicates the similarity of all taxa among different samples and the horizontal clustering indicates the similarity of certain taxa among different samples. Venn diagram can visually display the number of common and unique OTUs in multi-samples and groups. Core-Pan OTU analysis represents the intermediate circle that indicates the common OTU numbers of all the samples and the ellipse outside indicates the unique OTU numbers of different samples. The species accumulation (SA) analysis was performed to show the increase in OTUs with the addition of each sample. The COG function classification was performed using the software Phylogenetic Investigation of Communities by Reconstruction of Unobserved States (PICRUSt) in I-Sanger (http://www.i-sanger.com).

### Statistical Analyses

One-way ANOVA was used to examine the significance of alpha diversity indices. The samples belong to different groups, and the sample number in per group is more than 3, so we used the alpha diversity indices to analyze the differences among groups, and Kruskal-Wallis Test was used for multi-groups comparison. The indices were calculated by Mothur (v1.31.2), and the corresponding rarefaction curve was drawn by software R (v3.1.3) (Schloss et al., 2009). Analysis of molecular variance (AMOVA) was also performed by using Mothur, based on weighted-UniFrac distance (Schloss et al., 2009). The analysis of similarities (ANOSIM) and Adonis analyses were performed using vegan package of software R (v3.1.3) based on the Bray–Curtis distance metrics (Zhou et al., 2011). The ANOSIM and Adonis analysis using two different databases silva128/16s and green genes135/16s, with/without sub-sampling used the software R (v3.1.3) in I-Sanger (http://www.i-sanger.com). SILVA is a database of aligned small (16S/18S, SSU) and large subunit (23S/28S, LSU) rRNA sequences for Bacteria, Archaea, and Eukarya (www.arb-silva.de). The Greengenes provides database of small subunit ribosomal near-full length sequences from Bacteria and Archaea (http://greengenes.secondgenome.com). Metastats (http://metastats.cbcb.umd.edu/) and R (v3.1.3) were used to determine which taxonomic groups were significantly different between groups (four samples per group). We also adjusted the obtained *P*-value by a Benjamini-Hochberg false discovery rate correction [function “p.adjust” in the stats package of R (v3.1.3)] (Benjamini and Hochberg, 1995; White et al., 2009).

### Quantification of *nifH* by Quantitative Real-Time PCR

To compare the relative abundance of the *nifH* gene in different samples, a quantitative real-time PCR with a CFX connect real-time PCR System (Bio-Rad, USA) assay was performed. We used the primer pairs of PolF–PolR (Poly et al., 2001) for the *nifH* gene and 338f−518r (Yang et al., 2011) for the 16S rDNA as internal control. The reaction volume of qPCR containing 50 ng metagenomic DNA (~1 μL), 0.5 μL each primer (5 pM) and 10 μL of 2 × SYBR Green mixture (FastStart Universal Probe Master, Roche Life Science, Switzerland), then add water to reach final 20 μL volume. The PCR program was as follows: 95°C for 10 min followed by 40 cycles consisting of 95°C for 15 s and 60°C for 1 min. All qPCR reactions were run in three technical duplicates with each DNA sample, and each group contained four biological replicates. The melting curve was analyzed to assess *nifH* gene in each run. Finally, the relative gene level was calculated by using the 2^−Δ*CT*^method (Schmittgen and Livak, 2008). Significance analysis of q-PCR results was done using one-way analysis of variance (one-way ANOVA).

### Ethics Statement

The Ministry of Agriculture of the People's Republic of China issued permissions for the locations. The field studies did not involve endangered species. The experimental field was not protected or privately owned in any way.

## Results

### Basic Analysis of Soils and Plants

According to the information we got from China Soil Database (http://vdb3.soil.csdb.cn/), the soil type in Yuanyang, Xinxiang City belongs to fluvo-aquic soils and the soil type in Zhangqiu, Jinan City is calcic sajiang black soils which belong to lime concretion black soils. Then the samples were sent to the Center of Modern Analysis Nanjing University to measure the basic data of soils and plants. For soil analysis, the pH value, water content, carbon content and nitrogen content were tested. For plant analysis, the carbon content and nitrogen content were tested (Table 1). The soils we used for test is bulk soil which was far from the roots of plants. As shown in Table 1, the pH values were all about 8. Water content and carbon content of soils in Yuanyang at tasseling stage is higher than in Zhangqi at flowering stage. In conclusion, the soil analysis showed no significant difference between CC2 and Z958. However, the plant analysis showed significant difference between CC2 and Z958 in nitrogen content (Table 1).

**Table 1:** Soil analysis and plant analysis of CC2 and Z958.

	**Sampling stages**	**Tasseling stage (Mean** **±** **SD)**		**Flowering stage (Mean** **±** **SD)**	
	**Samples**	**CC2**	**Z958**	**CC2**	**Z958**
Soil analysis	pH value	8.00 ± 0.14	8.02 ± 0.18	7.97 ± 0.12	7.91 ± 0.12
	Water content (%)	17.38 ± 0.10	17.21 ± 0.11	15.36 ± 0.23	15.03 ± 0.37
	C content (%)	1.80 ± 0.14	1.79 ± 0.15	1.15 ± 0.19	1.25 ± 0.32
	N content (%)	0.08 ± 0.01	0.08 ± 0.01	0.10 ± 0.00	0.09 ± 0.02
Plant analysis	C content (%)	41.70 ± 0.30	42.21 ± 2.23	41.90 ± 0.16	41.38 ± 0.29
	N content (%)	3.51 ± 0.09	3.56 ± 0.26	3.01 ± 0.43^*^	2.22 ± 0.07^*^

*SD represents standard deviation. Values were mean ± SD (n = 4). CC2 and Z958 represent the transgenic maize CC-2 and its recipient cultivar Z958. T and F represent the tasseling and flowering stages. SS, Rh, and Rt represent the surrounding soil samples, rhizospheric soil samples and root samples, respectively. C content and N content represent carbon content and nitrogen content. The significance test was performed using one-way ANOVA. The values in asterisk indicate the significant difference (P < 0.05) between the CC2 and Z958 groups by the tests*.

### Comparative Analysis of the Alpha Diversity of Root-Associated Bacterial Communities Between CC2 and Z958

Supplementary Figure 1 shows the Venn diagram and Supplementary Figure 2 represents the Core-Pan OTU analysis. As shown in Supplementary Figure 1, the surrounding soil and the rhizospheric soil shared the largest amount of OTUs meanwhile the rhizospheric soil and root samples shared the minimum amount of OTUs. Furthermore, when compared the root microorganisms at same layers (surrounding soil samples, rhizospheric soil samples, and root samples), the results showed that the surrounding soil samples of CC2 and Z958 shared the largest amount of OTUs while the root samples of CC2 and Z958 shared the minimum amount of OTUs. Supplementary Figure 3A shows the curve obtained using all the OTU data and species evenness was derived from the slope of the line that fits the graph (Supplementary Figure 3B). The species accumulation (SA) analysis indicated that the abundances of different species were similar. The rarefaction curves of the observed OTU numbers of samples indicated that the sequencing depth and the OTU coverage of the samples included sufficient detectable species in bacterial communities and captured the diversity of bacterial communities (Supplementary Figure 4).

**Figure 1:**
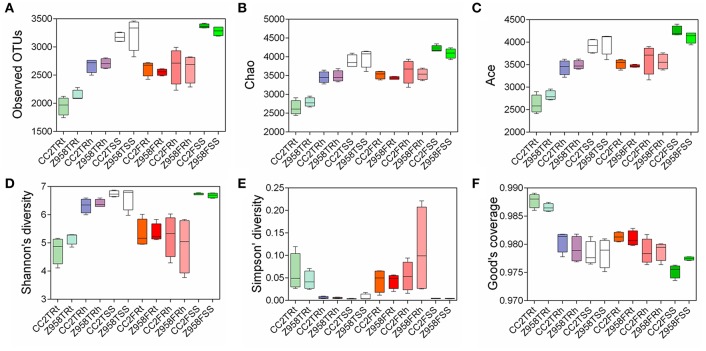
The boxplot of alpha diversity of observed OTUs value **(A)**, Chao value **(B)**, ACE value **(C)**, Shannon value **(D)**, Simpson value **(E)**, and Good's coverage **(F)**. Treatment's details were as in Table 1.

Alpha diversity was applied for analyzing complexity of species diversity for a sample through several indices (Schloss et al., 2009). The analysis of alpha diversity through six alpha diversity indices and the mean and SD were calculated based on the alpha diversity values of all the samples and Kruskal-Wallis Test was used for multi-groups comparison (Supplementary Figure 4). The results of alpha diversity through different indices and the values were calculated the mean and standard deviation (SD) of six alpha diversity indices, different groups divided in sampling stages and different groups divided in sampling compartments (Supplementary Table 4). We observed that all the groups divided in sampling compartments have significant difference in six indices of the alpha diversity (Supplementary Table 4), indicating that the sampling compartments have great influence on the composition of microbial community. Then a boxplot of alpha diversity was drawn (Figure 1), different groups were divided into sampling stages (Supplementary Figure 5A) and compartments (Supplementary Figure 5B). No significant difference in alpha diversity (i.e., all pair-comparison showed *p* > 0.05) of the surrounding soil, rhizospheric soil and root samples was found between CC2 and Z958 at the tasseling and flowering stages (Figure 1). And as shown in Supplementary Figure 5, the different boxplot of six alpha diversity indices that the species richness and evenness of the bacterial communities were different in different sampling stages and compartments.

### Comparative Analysis of the Beta Diversity of Root-Associated Bacterial Communities Between CC2 and Z958

**Figure 2:**
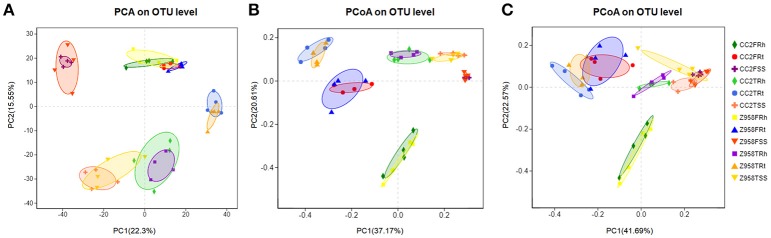
**(A)** PCA based on OTU abundance of bacterial communities. PCoA based on Bray-Curtis distance **(B)** and weighted-unifrac distance **(C)** of root-associated bacterial communities between CC2 and Z958. Treatment's details were as in Table 1.

The similarities of the composition among the samples determine the distance in the Figure 2A. Based on the OTU table for biom-format (Supplementary Table 5), the relative abundance of each OTU in each sample was calculated, and the principal component analysis (PCA) of OTU was done with the relative abundance value. First, we examine the differences in the OTU composition between the surrounding soil, rhizospheric soil and roots samples of CC2 and Z958 at the tasseling and flowering stages by PCA. As shown in Figure 2A, the samples at same developmental stage and compartments were clustered into one group, and there was no significant distinction in distance among different genotypes of maize. Similar results were obtained using the different PC1 and PC2 (Supplementary Figure 6). The bacterial communities in the surrounding soil, rhizospheric soil and roots samples of CC2 were not distinct from those of Z958 at the tasseling or flowering stage.

Then, beta diversity analysis was performed by principal coordinate analysis (PCoA) based on Bray-Curtis distance (Figure 2B) and weighted-unifrac distance (Figure 2C) of root-associated bacterial communities of CC2 and Z958. As shown in Figure 2, the root-associated bacterial communities from different compartments of CC2 were not distinct from those of Z958 at the tasseling or flowering stages. PCoA was also performed using another group and got similar results (Supplementary Figure 7).

Then we performed statistical analysis of similarities (ANOSIM) of bacterial communities. The ANOSIM based on the Bray–Curtis distance indicated that the beta diversity of bacterial communities in different sampling stages or compartments had no significant difference (Supplementary Table 6). Furthermore, the results of ANOSIM of indicated that the bacterial communities of CC2 and Z958 had no significant difference (Table 2; Supplementary Table 6), while the results of Adonis of all samples indicated that the bacterial communities of CC2 existed significant difference (*P* < 0.05) with that of Z958 in the surrounding soils at the tasseling stage (Table 2; Supplementary Table 7). Then ANOSIM and Adonis were conducted based on the weighted-unifrac distance metrics and indicated that there existed significant difference between CC2 and Z958 in the surrounding soils at the tasseling stage (Table 2).

**Table 2:** Statistical analyses of bacterial community structure between the maize line CC2 and its recipient cultivar Z958 with two different approaches.

**Distance metrics**	**Group vs. Group**	**Adonis**		**ANOSIM**	
		**F. Model**	***P-*value**	***R*-value**	***P*-value**
Bray–Curtis	CC2TSS vs. Z958TSS	2.408717	0.032967^*^	0.250	0.065
	CC2TRh vs. Z958TRh	0.873021	0.472527	−0.031	0.489
	CC2TRt vs. Z958TRt	1.777028	0.164835	0.333	0.052
	CC2FSS vs. Z958FSS	0.840308	0.492507	0.052	0.318
	CC2FRh vs. Z958FRh	0.215798	0.957043	−0.135	0.785
	CC2FRt vs. Z958FRt	1.298254	0.280719	0.135	0.276
weighted-unifrac	CC2TSS vs. Z958TSS	2.2614	0.026^*^	0.2708	0.037^*^
	CC2TRh vs. Z958TRh	0.75939	0.559	−0.0938	0.579
	CC2TRt vs. Z958TRt	1.7281	0.218	0.2708	0.105
	CC2FSS vs. Z958FSS	0.75651	0.559	0.1354	0.254
	CC2FRh vs. Z958FRh	0.26616	0.913	−0.125	0.661
	CC2FRt vs. Z958FRt	1.032	0.357	0.0104	0.382

ANOSIM and Adonis based on the Bray–Curtis and weighted-unifrac distance metrics. The P-values in bold indicate the significant difference [P < 0.05(^*^)] between the CC2 and Z958 groups by the tests. Treatment's details were as in Table 1.

In order to verify the results, the ANOSIM and Adonis were also performed based on the weighted-unifrac and unweighted-unifrac, and the results indicated that the phylogenetic beta diversity of the bacterial communities in the surrounding soils at the tasseling stage of CC2 were significantly different (*P* < 0.05) from that of Z958. Then different databases with/without sub-sampling (Supplementary Table 8) were used and got the similar results. As shown in Figures 2B,C, one replicate in Z958TSS and one replicate in CC2TSS had the largest difference among the groups, so we removed these two replicates and performed ANOSIM and Adonis again with different databases with/without sub-sampling (Table 3). As predicted, the taxonomic beta diversity of bacterial communities of CC2 and Z958 in the surrounding soils at the tasseling stage showed no significant difference via ANOSIM and Adonis.

**Table 3:** Significance tests of bacterial community structure between CC2TSS and Z958TSS with three replicates.

**Database**	**Treatment**	**Adonis**		**ANOSIM**	
		**F. Model**	***P-*value**	***R*-value**	***P*-value**
silva128/16s	Without subsampling	2.0956	0.1	0.5926	0.092
	With subsampling	2.2353	0.1	0.6296	0.1
greengenes135/16s	Without subsampling	2.0956	0.1	0.5926	0.099
	With subsampling	2.1184	0.1	0.6296	0.087

*ANOSIM and Adonis based on the Bray–Curtis distance metrics. Treatment's details were as in Table 1*.

The phylogenetic tree at the genus level was constructed using the bacterial communities to evaluate similarity in species composition among samples (Supplementary Figure 8). Cluster trees of beta diversity based on the Bray-Curtis distance (Supplementary Figure 8A), weighted-unifrac distance (Supplementary Figure 8B) and unweighted-unifrac distance (Supplementary Figure 8C). Heat map of beta diversity distance distribution is shown in Supplementary Figure 9. The samples at the same sampling stage and compartment were clustered into one group, and indicated that there was no obvious difference between different genotypes of maize.

### Comparison of the Composition of the Major Bacterial Taxa at Two Developmental Stages

The taxonomic composition distribution histogram of each sample was shown at phylum (Supplementary Figure 10A), class (Supplementary Figure 10C), order (Supplementary Figure 10B), family (Supplementary Figure 10D), genus (Supplementary Figure 10E), and species (Supplementary Figure 10F) level. The ratio of each species in a certain sample was directly displayed. At phylum level, all taxa were used to draw the histogram. The most abundant phylum was Proteobacteria followed by Acidobacteria, Bacteroidetes, Chloroflexi, and Actinobacteria in the surrounding soil samples meanwhile Proteobacteria was the most abundant phylum followed by Bacteroidetes, Acidobacteria, Firmicutes, and Actinobacteria in the rhizospheric soil samples. However, in the root samples, Proteobacteria, Actinobacteria, Cyanobacteria, Acidobacteria, and Bacteroidetes were the five major abundant phyla (Supplementary Table 9; Supplementary Figure 10A). The major bacterial phyla at the tasseling and flowering stages had no significant difference and there was also no significant difference between CC2 and Z958 at the same sampling stage and compartment. At species level, *Arthrobacteroxydans* was found to be the major abundant species in the surrounding soil and root samples, meanwhile *Arthrobacteroxydans* and *Roseatelesdepolymerans* were the major abundant species in the rhizospheric soil samples at the flowering stage but *Arthrobacteroxydans* and *Bacillusmuralis* were the major abundant species in the rhizospheric soil samples at the tasseling stage (Supplementary Table 9; Supplementary Figure 10F). Those species whose abundance was <0.5% in all samples were classified into “others” in other ranks. The composition of the bacterial community from different genotypes of maize only existed difference in the rhizospheric soil samples at the tasseling stage. Supplementary Figure 11 shows the heat map of cluster analysis of samples in phylum (A), class (B), order (C), family (D), genus (E), and species (F) levels. At phylum level, all taxa were used to draw the heat map. The samples from same sampling compartments were clustered at the horizontal axis, and the color of samples from same sampling compartments showed same trend at the longitudinal axis at different taxonomic ranks (Supplementary Figure 11).

Then we compared the composition of the major bacterial taxa at two developmental stages, and calculated the Wilcoxon Rank-Sum Test of the relative abundance of bacteria at the phylum, class, order or family levels (Supplementary Table 10). As shown in Table 4, at phylum level, the relative abundance of Chloroflexi was significantly lower in the roots of CC2 than that of Z985 at the tasseling stage. At class level, the relative abundance of Bacilli in the roots of CC2 was significantly lower while the relative abundance of Betaproteobacteria in the roots of CC2 was significantly higher than that of Z985 at the tasseling stage. At order level, the relative abundance of Bacillales in the roots of CC2 was significantly lower. Furthermore, the relative abundance of Burkholderiales in the roots of CC2 was significantly higher than that of Z985 at the tasseling stage. At family level, the relative abundance of *Bacillaceae* in the roots of CC2 was significantly lower; meanwhile the relative abundance of *Comamonadaceae* in the roots of CC2 was significantly higher than that of Z985 at the tasseling stage (Table 4; Supplementary Table 10).

**Table 4:** The mean of bacterial relative abundance at different taxonomic level (%).

		**CC2TRt**	**Z958TRt**	**CC2TRh**	**Z958TRh**	**CC2TSS**	**Z958TSS**	**CC2FRt**	**Z958FRt**	**CC2FRh**	**Z958FRh**	**CC2FSS**	**Z958FSS**
**Phylum**	Chloroflexi	**3.97**	**5.49**	6.14	5.88	7.60	7.06	5.12	5.06	3.04	3.15	6.45	6.58
**Class**	Bacilli	**1.67**	**3.83**	6.76	3.55	0.65	0.63	5.79	4.17	6.09	6.23	0.94	0.90
	Thermomicrobia	**0.05**	**0.13**	0.19	0.14	0.16	0.15	0.06	0.06	0.04	0.02	0.11	0.10
	Thermoleophilia	**0.46**	**0.76**	1.09	0.99	0.80	0.57	1.00	0.93	0.41	0.28	0.94	1.03
	Acidobacteriia	0.003	0.010	**0.02**	**0.04**	0.09	0.10	0.03	0.03	0.03	0.03	0.11	0.10
	Betaproteobacteria	**18.9**	**8.09**	9.06	7.55	9.71	7.39	14.0	9.16	9.25	7.77	7.11	6.73
	Gemmatimonadetes	0.15	0.20	1.07	0.86	2.80	1.96	**0.43**	**0.29**	0.73	0.71	1.40	1.52
**Order**	Bacillales	**1.63**	**3.82**	6.71	3.53	0.63	0.62	5.79	4.16	6.0	6.23	0.94	0.90
	Gaiellales	**0.22**	**0.36**	0.46	0.46	0.36	0.27	0.63	0.58	0.26	0.18	0.64	0.70
	Rhodobacterales	**0.18**	**0.25**	0.50	0.56	0.60	0.60	0.23	0.19	0.25	0.21	0.43	0.52
	Oceanospirillales	**0**	**0.01**	0.008	0	0.02	0.003	0.004	0.013	0.001	0.005	0.017	0.004
	Acidobacteriales	0.003	0.010	**0.025**	**0.048**	0.09	0.10	0.03	0.03	0.03	0.03	0.11	0.10
	Methylophilales	0.17	0.22	**0.06**	**0.19**	0.08	0.12	0.22	0.40	0.78	0.67	0.09	0.10
	Burkholderiales	**18.33**	**7.30**	5.88	4.61	3.06	3.36	12.7	7.91	6.40	4.99	2.49	2.01
	Marinicellales	0.00	0.001	0.008	0.017	0.011	0.009	**0.003**	**0.011**	0.007	0.010	0.02	0.02
	Legionellales	0.22	0.16	0.17	0.26	0.32	0.28	**0.15**	**0.11**	0.15	0.15	0.39	0.39
**Family**	*Bacillaceae*	**1.11**	**3.34**	5.85	3.07	0.46	0.38	4.46	2.97	4.60	4.60	0.62	0.62
	*Gaiellaceae*	**0.21**	**0.35**	0.45	0.44	0.35	0.27	0.62	0.57	0.25	0.17	0.63	0.69
	*Intrasporangiaceae*	**0.14**	**0.23**	0.17	0.16	0.115	0.08	0.15	0.13	0.03	0.03	0.05	0.04
	*Methylobacteriaceae*	**0.009**	**0.051**	0.01	0.01	0.001	0.003	0.012	0.006	0.004	0.001	0.001	0.003
	*Nocardioidaceae*	**1.07**	**1.48**	1.04	1.21	0.51	0.46	0.57	0.83	0.28	0.25	0.36	0.34
	*Halomonadaceae*	**0**	**0.013**	0.008	0	0.02	0.003	0.004	0.013	0.001	0.005	0.017	0.004
	*Koribacteraceae*	0.003	0.010	**0.02**	**0.04**	0.09	0.10	0.03	0.03	0.03	0.03	0.11	0.10
	*Legionellaceae*	0.009	0.014	**0.003**	**0.014**	0.008	0.004	0.004	0.003	0.000	0	0.005	0.004
	*Methylophilaceae*	0.17	0.22	**0.06**	**0.19**	0.08	0.12	0.22	0.40	0.78	0.67	0.09	0.10
	*Comamonadaceae*	**17.61**	**6.52**	4.97	3.87	2.38	2.64	11.11	6.02	4.23	3.42	1.69	1.38
	*Turicibacteraceae*	0.02	0.008	**0.05**	**0.02**	0.01	0.01	0.001	0	0	0	0	0
	*Aurantimonadaceae*	0.55	0.59	0.27	0.25	0.05	0.04	**0.10**	**0.13**	0.05	0.06	0.01	0.02
	*Burkholderiaceae*	0.01	0.01	0.01	0.01	0.005	0.007	**0.007**	**0.019**	0.01	0.01	0.01	0.005
	*Marinicellaceae*	0.000	0.001	0.008	0.017	0.011	0.009	**0.003**	**0.011**	0.007	0.010	0.02	0.02
	*Sporichthyaceae*	0.003	0.004	0.02	0.01	0.02	0.01	**0.009**	**0.016**	0.012	0.006	0.01	0.01
	*Actinosynnemataceae*	0.43	0.49	0.22	0.13	0.15	0.07	**0.28**	**0.38**	0.04	0.07	0.04	0.02
	*Alcaligenaceae*	0.12	0.12	0.12	0.15	0.12	0.09	**0.12**	**0.07**	**0.21**	**0.11**	0.36	0.35
	*Alicyclobacillaceae*	0.01	0.02	0.14	0.06	0.03	0.05	**0.08**	**0.04**	0.01	0.02	0.04	0.03
	*Coxiellaceae*	0.17	0.11	0.11	0.18	0.21	0.18	**0.10**	**0.08**	0.11	0.12	0.27	0.29
	*Amoebophilaceae*	0	0.00	0.00	0	0	0.003	**0.007**	**0.000**	0.001	0.001	0.003	0.003

*The mean of bacterial relative abundance at phylum, class, order or family levels. Bold letters show significant difference between CC2 and Z958 calculated by Wilcoxon Rank-Sum Test. Treatment's details were as in Table 1*.

As shown in Table 4, the relative abundances of Betaproteobacteria, Burkholderiales and Comamonadaceae were significant higher in the roots of CC2 than that of Z985 at the tasseling stage; meanwhile the relative abundances of Burkholderiales and Comamonadaceae were also higher in the roots than in the rhizospheric soil and surrounding soil at both two stages. In order to verify their Enrichments/Derichments situations at the genus level, he relative abundance of different genera belonging to the family *Comamonadaceae* was compared (Table 5). As shown in Table 5, the relative abundance of *Variovorax* in the roots of CC2 was significantly lower; while *Acidovorax* in the roots of CC2 was significantly higher than that of Z985 at the tasseling stage. And the relative abundance of *Hylemonella* was significantly higher than that of Z985 at the flowering stage. *Acidovorax* was enriched in the roots than in the rhizospheric soil and surrounding soil at both two stages, particularly in root samples of CC2 at the tasseling stage.

**Table 5:** The mean of bacterial relative abundance of main genera belonging to the family Comamonadaceae (%).

**GENUS**	**CC2TRt**	**Z958TRt**	**CC2TRh**	**Z958TRh**	**CC2TSS**	**Z958TSS**	**CC2FRt**	**Z958FRt**	**CC2FRh**	**Z958FRh**	**CC2FSS**	**Z958FSS**
*Rubrivivax*	1.06	0.71	0.83	0.89	0.52	0.52	1.11	0.89	0.68	0.66	0.53	0.46
*Methylibium*	1.68	0.51	0.52	0.46	0.14	0.18	0.60	0.53	0.21	0.22	0.13	0.10
*Comamonas*	0.00	0.04	0.00	0.00	0.00	0.00	0.00	0.00	0.00	0.01	0.00	0.00
*Hydrogenophaga*	0.05	0.11	0.03	0.15	0.02	0.12	0.01	0.02	0.02	0.02	0.02	0.01
*Ramlibacter*	0.24	0.19	0.65	0.41	0.57	0.78	0.80	0.60	0.70	0.57	0.41	0.33
*Variovorax*	**0.29**	**0.53**	0.36	0.33	0.14	0.12	0.03	0.05	0.03	0.03	0.03	0.03
*Polaromonas*	0.00	0.00	0.02	0.01	0.01	0.00	0.00	0.00	0.00	0.01	0.00	0.00
*Pelomonas*	0.08	0.07	0.07	0.07	0.03	0.06	0.08	0.08	0.10	0.12	0.03	0.01
*Inhella*	0.00	0.00	0.00	0.00	0.00	0.14	0.00	0.00	0.00	0.00	0.01	0.00
*Hylemonella*	0.11	0.12	0.26	0.20	0.17	0.22	**0.23**	**0.17**	0.32	0.32	0.18	0.17
*Roseateles*	3.38	1.40	0.58	0.24	0.21	0.20	6.59	2.76	1.03	0.37	0.10	0.07
*Acidovorax*	**10.47**	**2.77**	1.49	1.01	0.45	0.18	1.30	0.59	0.44	0.28	0.07	0.04

*Those relative abundances with bold letters show significant difference between CC2 and Z958 calculated by Wilcoxon Rank-Sum Test. Treatment's details were as in Table 1*.

### Comparison of the Composition of Main Nitrogen-Fixing/Phosphate-Solubilizing Bacterial Genera and the Relative Abundance of *nifH* Gene at Different Developmental Stages

**Figure 3:**
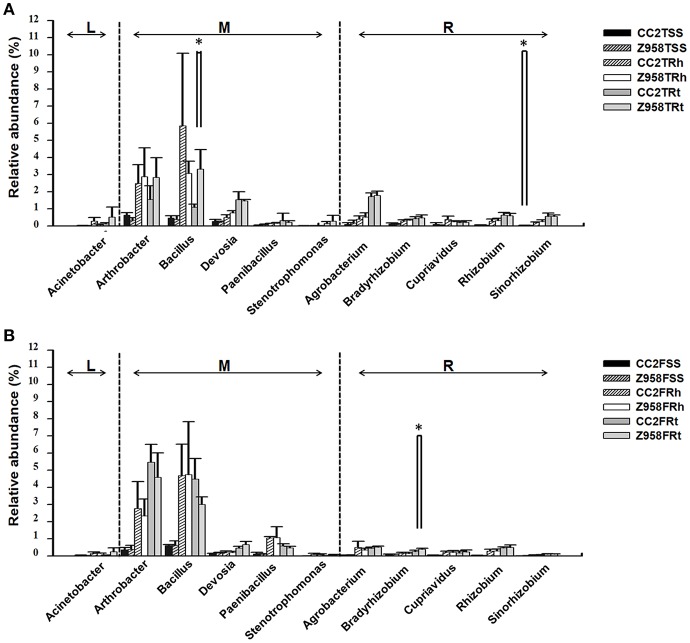
Relative abundances of main nitrogen-fixing bacterial genera at tasseling **(A)** and flowering stages **(B)**. Treatment's details were as in Table 1. L, M, and R represent the nitrogen-fixing bacterial genera belonging to free-living nitrogen-fixation, associative nitrogen-fixation and symbiotic nitrogen-fixation, respectively. Values are mean ± SD (*n* = 4). Asterisk (^*^p < 0.05) indicates significant difference according to Wilcox test.

Two hundred sixty-three genera were identified in the surrounding soil, rhizospheric soil and root samples of CC2 and Z958 at the tasseling and flowering stages (Supplementary Table 11). We compared the relative abundances of 11 main nitrogen-fixing bacterial genera at different developmental stages (Figure 3; Supplementary Tables 9, 10). At both the tasseling and flowering stages, there were no remarkable differences in the relative abundances of all nitrogen-fixing bacterial genera (Figure 3). However, the relative abundance of *Bacillus* in the root samples and *Sinorhizobium* in the surrounding soil at tasseling stage (Figure 3A), the relative abundance of *Bradyrhizobium* in the root samples at flowering stage (Figure 3B) shown significant difference between CC2 and Z958. Meanwhile, the relative abundances of 7 main phosphate-solubilizing bacterial genera (*Bacillus, Bradyrhizobium, Agrobacterium, Arthrobacter, Serratia, Flavobacterium, and Streptomyces*) were also compared at different developmental stages, and found that the relative abundance of *Bacillus* at tasseling stage and *Bradyrhizobium* at flowering stage were significantly lower in CC2 roots than that of Z958 roots (Supplementary Table 10).

**Figure 4:**
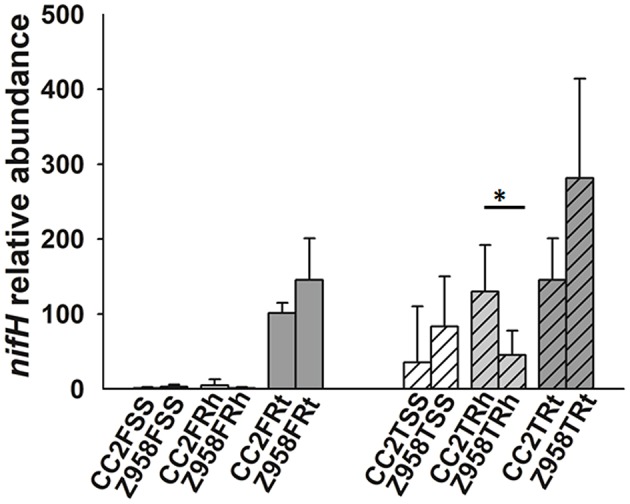
Relative abundance of *nifH* gene in maize root-associated bacterial communities of CC2 and Z958 at different sampling compartments and stages. Treatment's details were as in Table 1. Value of 1 was assigned to the detected value of the surrounding soil (rhizosphere part) of CC2 at the flowering stage (CC2FSS). The error bars represent the standard deviation of four replicates of soil or root samples and each replicate with technical triplicate. Asterisk (^*^p < 0.05) indicates significant difference according to Wilcoxon Rank-Sum Test.

Then the relative abundance of nitrogen-fixing or phosphate-solubilizing bacteria were compared at the class, order or family levels. As mentioned above, the relative abundances of class Bacilli, order Bacillales and the family *Bacillaceae* were significantly lower in the roots of CC2 than that of Z985 (Table 4; Supplementary Table 10). To further verify the results, we determined the copy number of *nifH* gene by qPCR assay (Figure 4), and found that the relative abundance of the *nifH* gene in most samples had no significant difference (Figure 4). The *nifH* gene abundance of surrounding soil and rhizospheric soil samples were at low levels compared to root samples. However, the relative abundance of the *nifH* gene in the rhizospheric soil of CC2 at tasseling stage was significantly higher than that of Z958 (Figure 4), and these findings were different from the results of 16S rDNA (V3-V4 region) amplicon deep sequencing showed by the relative abundances of main nitrogen-fixing bacterial genera (Figure 3).

## Discussion

We provided an overall framework of this study through comparison of the previous opinions on the concept of root-associated microbiota (Edwards et al., 2015; Rascovan et al., 2016; Lu et al., 2017, 2018). According to Edwards et al. the root-associated microbial communities include three compartments: rhizosphere, rhizoplane, and endosphere (Edwards et al., 2015). In our study, we adopted the surrounding soil, rhizospheric soil, and intact roots. The surrounding soil is which loosely adhering to the plant roots, and can easily be shaken off from the roots. The rhizospheric soil is tightly adhering to the plant roots, and can be washed off from the roots with PBS buffer (Inceoglu et al., 2010; Lu et al., 2018). The intact root samples include the rhizoplane and endosphere compartments. The rhizoplane at the root surface can only be sonicated and the endosphere is the microbial habitat inside the plant root i.e., the interior compartment (Bulgarelli et al., 2013; Edwards et al., 2015).

For the analysis of soil types under experimental cultivation, it was found that the sampling fields of Maize at tasseling and flowering stages belonged to fluvo-aquic soils and lime concretion black soils, respectively. Moreover, there was no significant difference in soil physical and chemical properties between the transgenic GT maize CC2 and its recipient variety Z958 in each period. This is also ensure that our subsequent experimental comparisons are in a single variable situation. However, the plant analysis showed the nitrogen content of Z958 was significant lower than that of CC2. According to our laboratory's long-standing experimental records, with the maturity of maize, the N content of plant will gradually decreased. Therefore, the low N content of Z958 may be due to the inconsistent growth rate with that of CC2.

In this study, our main interest was to find the potential impacts of transgenic GT maize on root-associated microbial communities related to genetic modified (GM) or transgenic crop biosafety evaluation in China, which is directly related to possibility of potential application. Bulgarelli et al. hypothesized that the species or genotypes of plant were the major factors for determining the composition and structure of root-associated bacterial communities (Bulgarelli et al., 2013). Thus, we initiated this project to evaluate the potential effects of the *EPSPS* transgenic maize line CC2 on the bacterial communities in the root-associated soil habitats compared with its recipient cultivar Z958. Previous studies hypothesized that the horizontal gene transfer, product of transgene expression (Dunfield and Germida, 2004), or root exudates (Liu et al., 2005; Bais et al., 2006) influence rhizosphere microbes.

In this study, no obvious difference in alpha diversity was found between root-associated microbial communities of CC2 and Z958. ANOSIM and Adonis were used to assess the statistical significance of microbial communities' diversity. As shown in Table 2, the result of Adonis showed that the bacterial communities in the surrounding soil at tasseling stage existed significant difference while ANOSIM showed no significant difference. This may be due to the high variations among technical replicates which are most likely affected by sampling artifacts associated with random sampling (Zhou et al., 2008, 2011). Zhou et al. assumed that these variations in technical replicates can lead to overestimating beta diversity of microbial communities, but less on alpha diversity (Zhou et al., 2011). As Zhou et al. mentioned that increasing biological replicates provide better resolution in differentiating the treatment effects, we removed one replicate in each group of surrounding soil with the largest difference among the group then there was no significant difference via analysis of ANOSIM and Adonis between CC2TSS and Z958TSS (Table 3). In order to verify the results of beta diversity, the composition of major bacterial phyla at two stages were compared and found that there were no significant difference between CC2 and Z958. The previous reports showed that the most abundant phylum in the root-associated microbial communities of soybean and in the rhizosphere soil of maize is Proteobacteria (Li et al., 2014; Rascovan et al., 2016; Lu et al., 2018), which is consistent with our present study.

The genus *Acidovorax* belongs to the family *Comamonadaceae* within the order Burkholderiales, and it includes 15 recognized species which were isolated from soil, water, activated sludge, clinical sources and infected plants (Willems et al., 1990; Chun et al., 2017). These species belong to pathogenic bacteria, saprophytic bacteria or plant-growth-promoting rhizobacteria (PGPR) (Han et al., 2016). Due to the sequencing depth and the limited length of 16S rDNA (V3-V4) amplicon, it is difficult to identify those OTUs at species level which belongs to the genus *Acidovorax*. However, the enrichment of *Acidovorax* in transgenic maize CC2 in root samples at tasseling stage was still worthy of attention.

This study also focused on the composition of nitrogen-fixing and phosphate-solubilizing bacteria. The relative abundance of nitrogen-fixing or phosphate-solubilizing bacteria in the rhizosphere and root showed the class Bacilli, the order Bacillales and the family *Bacillaceae* to be enriched in the roots and rhizospheric soil than in the surrounding soil, and significantly lower in the roots of CC2 than that of Z985. Then the relative abundances of 11 main nitrogen-fixing bacterial genera were compared and found some differences between the two maize lines. As shown in Figure 3, the relative abundance of *Sinorhizobium* in the surrounding soil at tasseling stage and *Bradyrhizobium* in the root samples at flowering stage showed significant differences, however, their abundance were very low, so it can't be assumed that root-associated nitrogen-fixing bacteria have been affected by the transgenic maize line CC2. The relative abundance of *Bacillus* in the root samples of CC2 was significantly lower than that of Z958 (Figure 3) at the tasseling stage, however, the relative abundance of the *nifH* gene in the root samples showed no significant difference (Figure 4). The relative abundances of 7 main phosphate-solubilizing bacterial genera were also compared and found that the relative abundances of *Bacillus* and *Bradyrhizobium* showed significant difference in the roots, depicting that the *EPSPS*-transgenic maize line CC2 affects not only nitrogen cycling but also phosphorus cycling. Some species of *Bacillus* belong to the PGPR or associated nitrogen-fixing bacteria (Lugtenberg and Kamilova, 2009). When focused on analyzing species level (Supplementary Table 10), the Wilcoxon Rank-Sum Test of main bacterial species showed that the relative abundances of *Bacillus cereus* and *Bacillus muralis* existed significant difference in the root samples at the tasseling stage, both of them belongs to PGPR class and have nitrogen-fixing and phosphate-solubilizing functions (Supplementary Table 10). Then we sum up the Wilcoxon Rank-Sum Test for different trait of samples at the tasseling and flowering stages (Supplementary Table 10), in the root samples, the relative abundance of single genus or species with significant difference was all lower in CC2 than that of Z958. This finding grabbed our attention, i.e., the genetically modified maize line CC2 affects the N and P cycles of soil microorganisms, especially root microorganisms which are closely related to plants. Moreover, the *nifH* gene abundance at flowering stage was lower than that at tasseling stage. This might be attributed to the effects of different locations on root-associated microbial communities (Picard et al., 2008). Furthermore, according to two-step selection model for root microbiota differentiation, Bacteroidetes and Firmicutes are enriched at roots (Bulgarelli et al., 2013), and *Bacillus* and *Bradyrhizobium* belong to Firmicutes and Bacteroidetes, respectively. Therefore, we hypothesized that the root of maize had an enrichment effect on nitrogen-fixing bacteria. The relative abundance of nitrogen-fixing/phosphate-solubilizing bacteria only showed differences in root samples between CC2 and Z958, and it's more likely that the effect of plants on root-associated bacteria is not direct enough or the duration is not long enough and may take years to track. Furthermore, the relative abundance of *nifH* gene in the root samples of CC2 at tasseling stage was lower than that of Z958, but there was no significant difference in statistics. The *nifH* gene abundance in the rhizospheric soil of CC2 was significantly higher than Z958 at the tasseling stage (Figure 4); the reason is still unknown and remains to be further studied. We verified by calculating the sum of relative abundances of main nitrogen-fixing bacterial genera (Supplementary Table 9) and species (Supplementary Table 10) and found that the sum of relative abundances of main nitrogen-fixing bacteria of CC2TRh was higher than that of Z958TRh. Although the differences between the relative abundances of main nitrogen-fixing bacterial genera and *nifH* gene are not entirely consistent, this might be due to many complex factors, such as the use of maize plants with water treatment, culture-independent amplicon deep sequencing of each sample. We sum up the Wilcoxon Rank-Sum Test of different trait of samples at different compartment (Supplementary Table 10), the alpha diversity (ACE index) of surrounding soil was much higher than that of rhizospheric soil and root samples while the relative abundance of nitrogen-fixing/phosphate-solubilizing bacteria in two stages of surrounding soil was much lower than that of rhizospheric soil and root samples. However, the relative abundance of *Bacillus* was significantly lower in root samples than that of rhizospheric soil of CC2, the reduction of the relative abundance of *Bacillus* in root samples of CC2 may be explained the result that the relative abundances of *Bacillus* in the root of CC2 were significantly lower than that of Z985. According to Edwards et al. the two genera shown significant difference, *Bacillus* and *Bradyrhizobium* were mainly located in the rhizoplane and endosphere of rice roots (Edwards et al., 2015), and the main nitrogen-fixing bacterial genera in the roots of rice were associative nitrogen-fixation bacteria, just like maize in this study. The relative abundances of *nifH* gene in the surrounding and rhizospheric soils were lower than that in the root samples, this may be due to the reason that, some harmful bacteria could be isolated outside the layer of endophytes and enriched some plant growth-promoting bacteria (PGPB), then cause the increase of the proportion of *nifH* genes (Compant et al., 2010; Kuan et al., 2016; Naylor et al., 2017).

Yan et al. reported that the enrichment processes in the rhizosphere select microbes with particular functional genes (Yan et al., 2017), then the COG function classification and the relative abundance of COG function classification were compared by using PICRUSt. Although there existed some bias by using this type of analysis (PICRUSt), it is helpful to elucidate whether there is functional selection between different plants or between different sampling compartments. As shown in Supplementary Figure 12, there was no significant separation between CC2 and Z958, and no significant separation between different sampling compartments. However, we cannot rule out whether there are differences in functional genes among samples. Then we make comparison of functional genes like COG1348, COG4656, COG5420, COG5456, and COG5554, which were directly related to nitrogen fixation by COG annotation (Supplementary Table 12). The results showed that there existed significant difference between different sampling compartments, especially between samples of rhizospheric soil and root samples. Moreover, there existed no significant difference between samples of CC2 and Z958 in same stages and same compartments. These results above are not entirely consistent with quantification of *nifH* by qPCR, this may due to the lack of detailed mapping of gene function by COG, and also prove that the quantitative analysis of nitrogen-fixing-related genes still needs comparison of multiple data and comprehensive consideration.

As shown in previous studies, the 16S rDNA amplicon deep sequencing has a low reproducibility for beta diversity analysis (Zhou et al., 2011, 2013). Thus, we adopted four sample replicates of different compartments of CC2 and Z958 in this study, because increasing sampling efforts and sample replicate number is the most effective way to improve technical reproducibility and quantitation (Zhou et al., 2013) that also have been confirmed by previous studies (Liang et al., 2014; Sugiyama et al., 2014; Lu et al., 2017, 2018). In this study, a total of 3,804,059 qualified paired-end clean reads with an average count of 79,251 per sample was obtained to achieve the desired 90% OTUs overlap (Zhou et al., 2013).

Previous reports showed that the *EPSPS*-transgenic plant Quest canola has a lower diversity in culturable root-endophytic bacterial community compared to the conventional Excel canola (Dunfield and Germida, 2001). Lopes *et al*. have reported that there existed difference between non-transgenic and transgenic soybean in the composition and diversity of culturable endophytic bacterial population (Lopes et al., 2016). *Bacillus thuringiensis* (Bt) has been globally used in pest control in maize (Singh and Dubey, 2016), however, Coz et al. reported no effects on soil microbial enzymes and properties while Chen et al. hold the opposite points (Coz et al., 2008; Chen et al., 2012). Xue et al. reported that gram-positive to gram-negative bacteria ratio was lower for soils with Bt maize compared to non-Bt maize (Xue et al., 2005). And the effects of proteins Cry in AMF is still ambiguous though the Bt proteins expressed in roots of most Bt maize lines (Saxena et al., 2004; Icoz and Stotzky, 2008). Furthermore, according to Wang et al., no significant differences are found in the abundance and community structure of archaea between the transgenic maize containing *cry1Ab* and *EPSPS* genes and its parental maize (Wang et al., 2017). This study can also provide ecological safety data for transgenic herbicide-resistant maize with *EPSPS* gene.

The root-associated microbiome is complex and composed by several different component mainly contains bacteria and fungi and have complex interaction between them (Duran et al., 2018). They can also interact with plants and form complex ternary structures. Our future project is to take into account the complex interaction among bacteria, fungi and plants, and evaluated on the entire community. Overall, all results indicated that the transgenic maize line has no significant impact on the bacterial community composition in the field. Individual significant changes occur at specific stages and compartments, which may be due to interaction with environmental factors rather than direct effects of GM maize. Generally, even if there is no direct evidence that the *EPSPS* transgenic GT maize affects the composition of the root-associated bacterial communities, we still need to remain vigilant and cautious, and carry out further researches.

## Conclusion

In this study, our results indicated that the *EPSPS*-transgenic maize line CC2 exerted no significant effects on the alpha and beta diversity of rhizosphere bacterial communities at the tasseling and flowering stages compared to its recipient cultivar Z958, meanwhile the *nifH* gene abundance and the relative abundances at class, order, family, genus or species level demonstrated that the root of maize had an enrichment effect on nitrogen-fixing or phosphate-solubilizing bacteria. However, there existed differences in the relative abundances of several main nitrogen-fixing and phosphate-solubilizing bacterial genera between root samples of CC2 and those of Z958, indicating that the transformation of *EPSPS* gene might affect some root-associated bacteria related to nitrogen and phosphorus cycling during field growth. Furthermore, the transgenic maize CC2 had an enrichment effect on the abundance of genus *Acidovorax* in the root samples at the tasseling stage. The reason for derichment of *Bacillus* and *Bradyrhizobium* or enrichment of *Acidovorax* in the root of transgenic maize CC2 are worthy of consideration for the future studies. Actually, our results showed that the cultivation of genetically modified maize, generally, did not have a significant impact on the overall soil bacterial community structure, although there existed some differences in certain rhizosphere microbial abundance between GM and non-GM maize.

## Author Contributions

Y-HY, G-HL, and J-LQ conceived and designed the experiments. Z-LW, M-KY, M-HD, Z-ZZ, Y-TL, and G-HW performed the experiments. Z-LW, M-KY, AF, X-MH, and R-WY did data analysis. C-HM, S-FY, and YZ contributed to resources. Z-LW and M-KY wrote the draft of manuscript. Y-HY, G-HL, and ZH contributed to review and edit the manuscript.

### Conflict of Interest Statement

The authors declare that the research was conducted in the absence of any commercial or financial relationships that could be construed as a potential conflict of interest.
