# Supplementary figures and images for: Enrichments/Derichments of Root-Associated Bacteria Related to Plant Growth and Nutrition Caused by the Growth of an EPSPS-Transgenic Maize Line in the Field
Source: Front Microbiol. 2019 Jun 18;10:1335. doi: 10.3389/fmicb.2019.01335 (PMC6591461; doi:10.3389/fmicb.2019.01335)

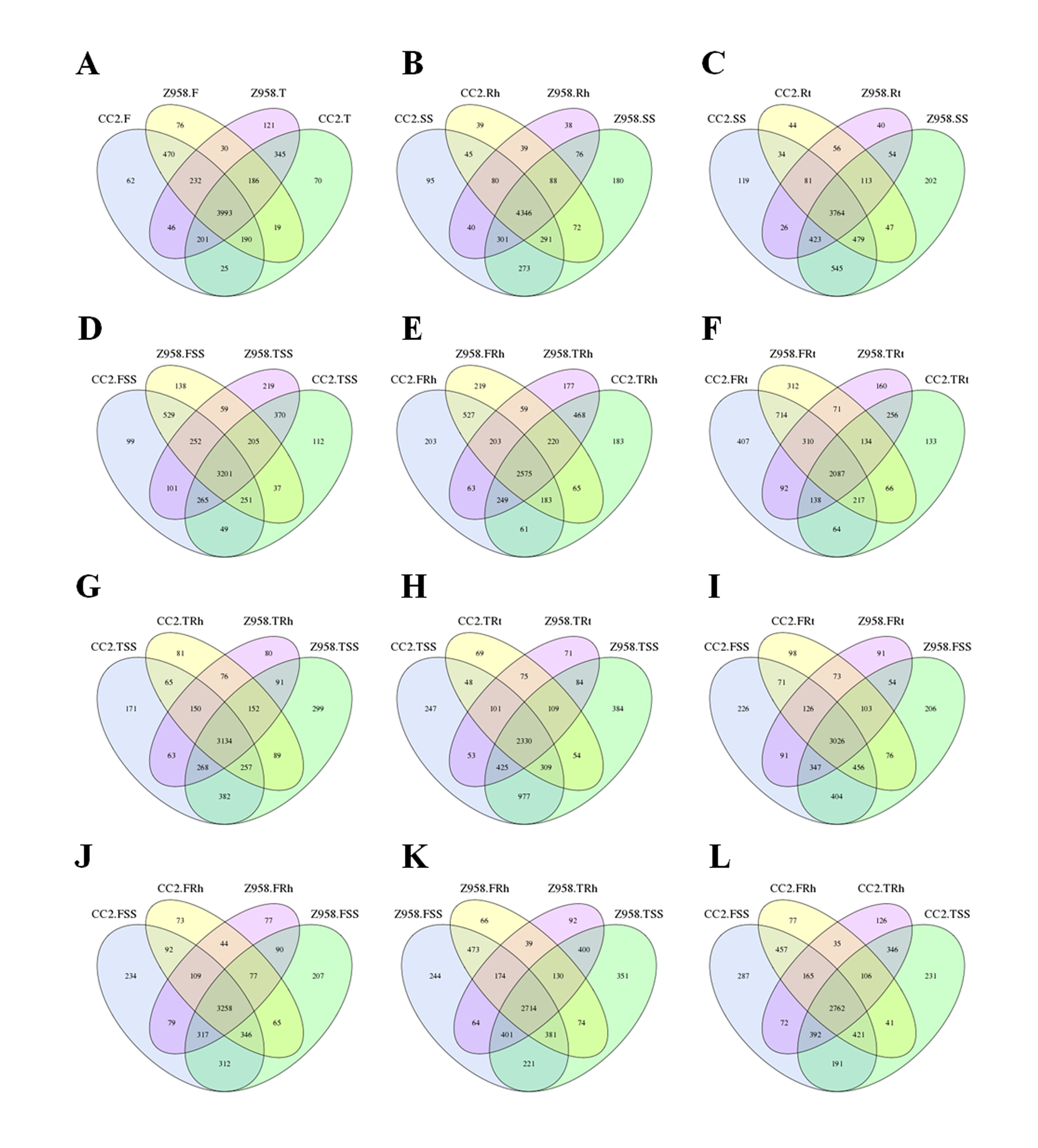

Supplement: Supplementary file 1 [file Image_1.TIF]

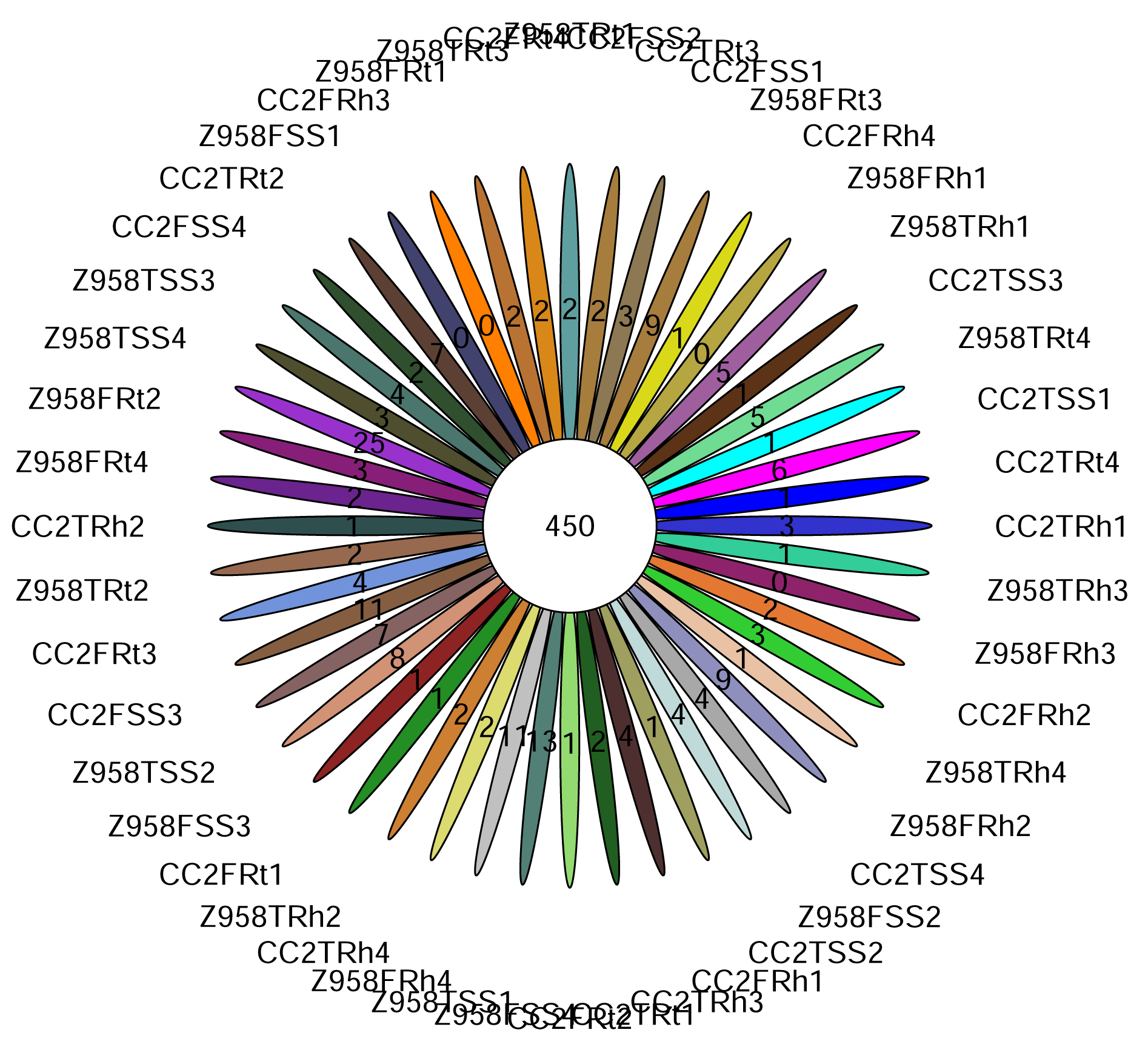

Supplement: Supplementary file 2 [file Image_2.TIF]

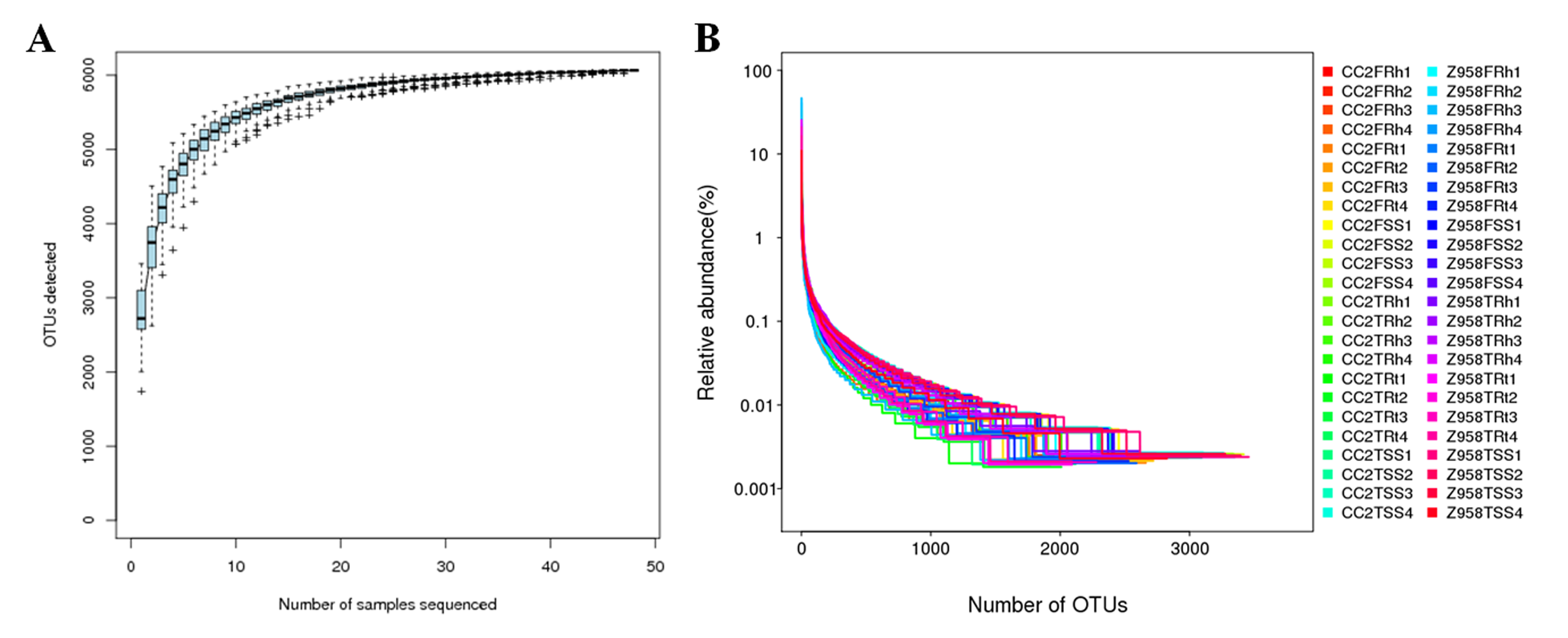

Supplement: Supplementary file 3 [file Image_3.TIF]

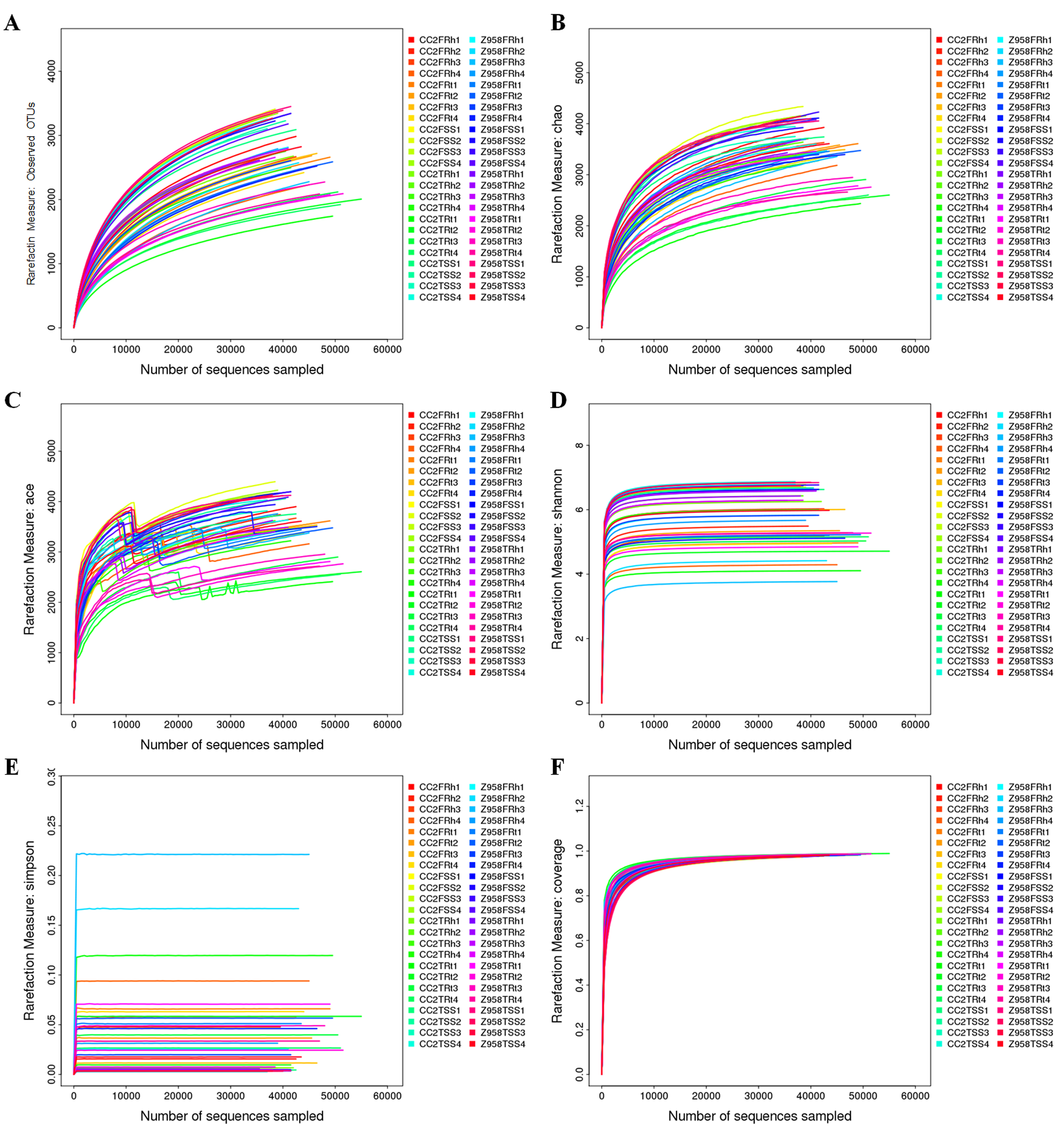

Supplement: Supplementary file 4 [file Image_4.TIF]

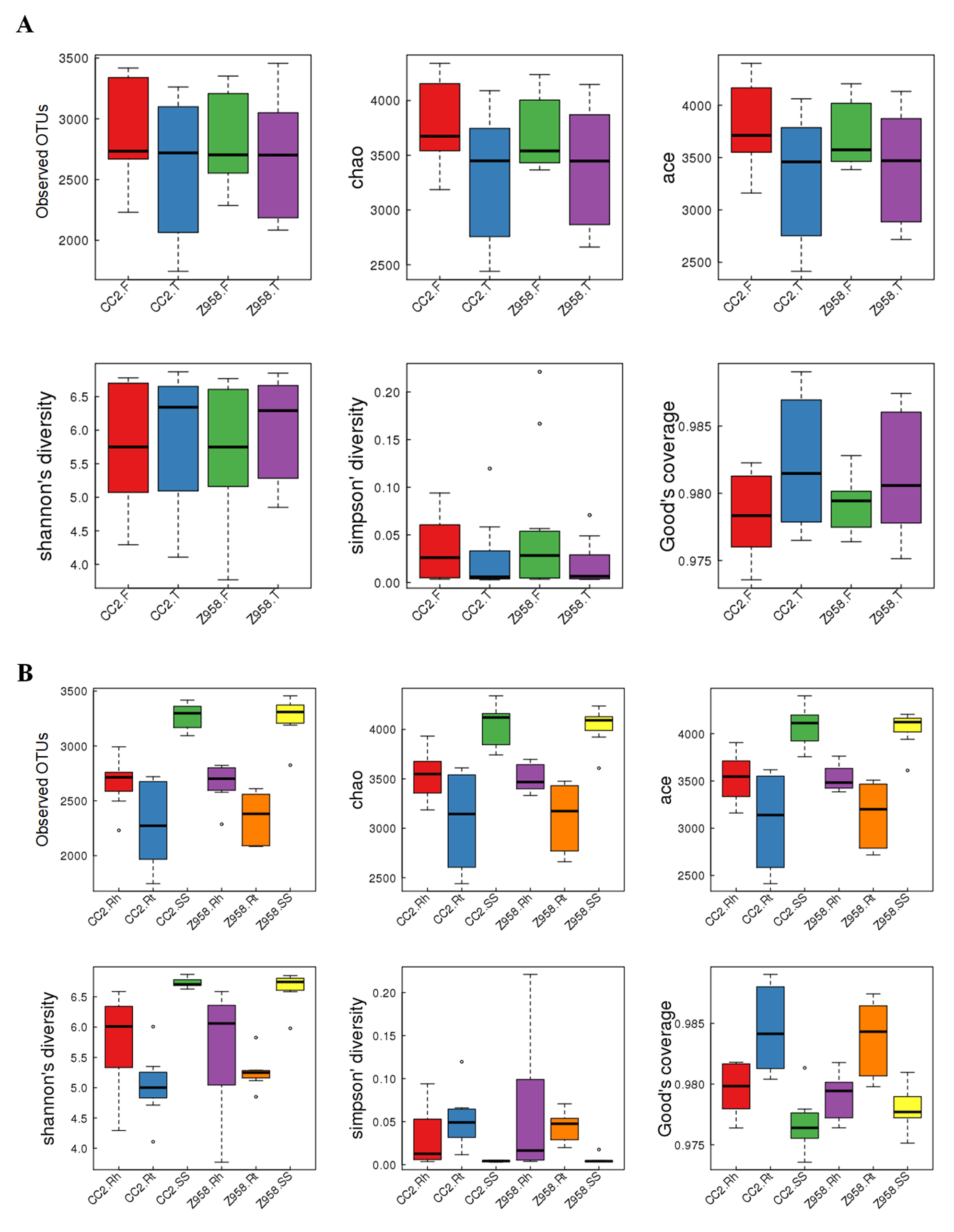

Supplement: Supplementary file 5 [file Image_5.TIF]

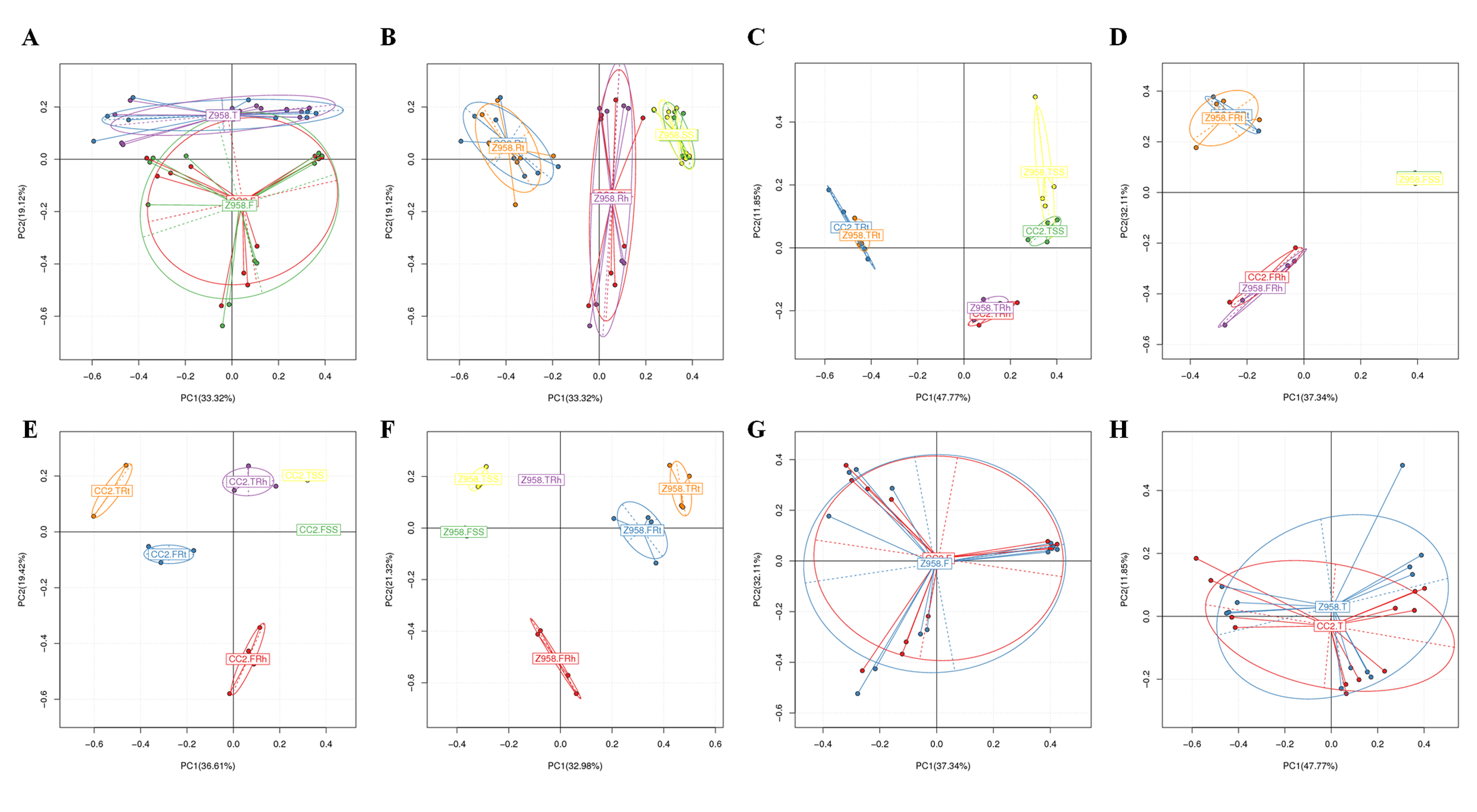

Supplement: Supplementary file 6 [file Image_6.TIF]

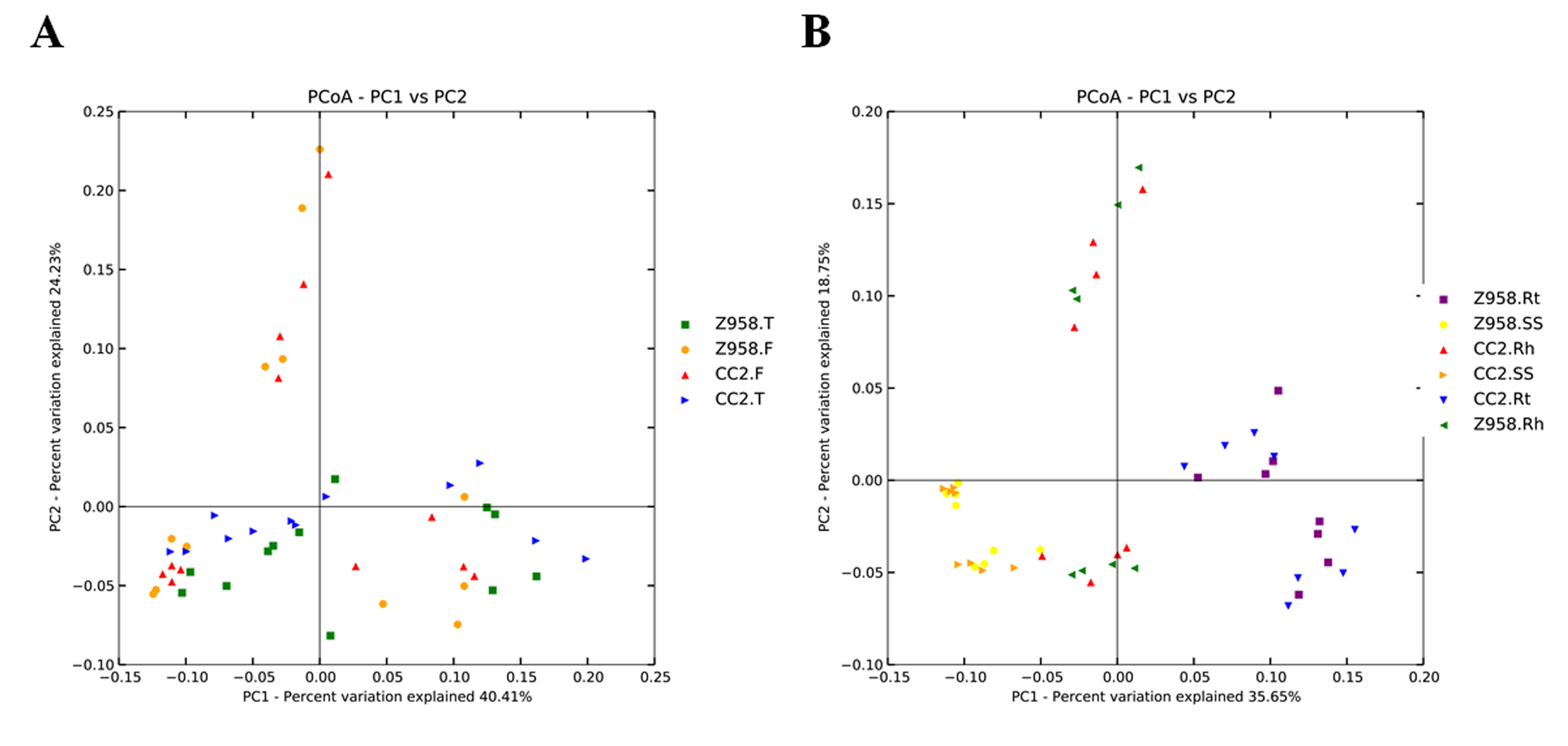

Supplement: Supplementary file 7 [file Image_7.TIF]

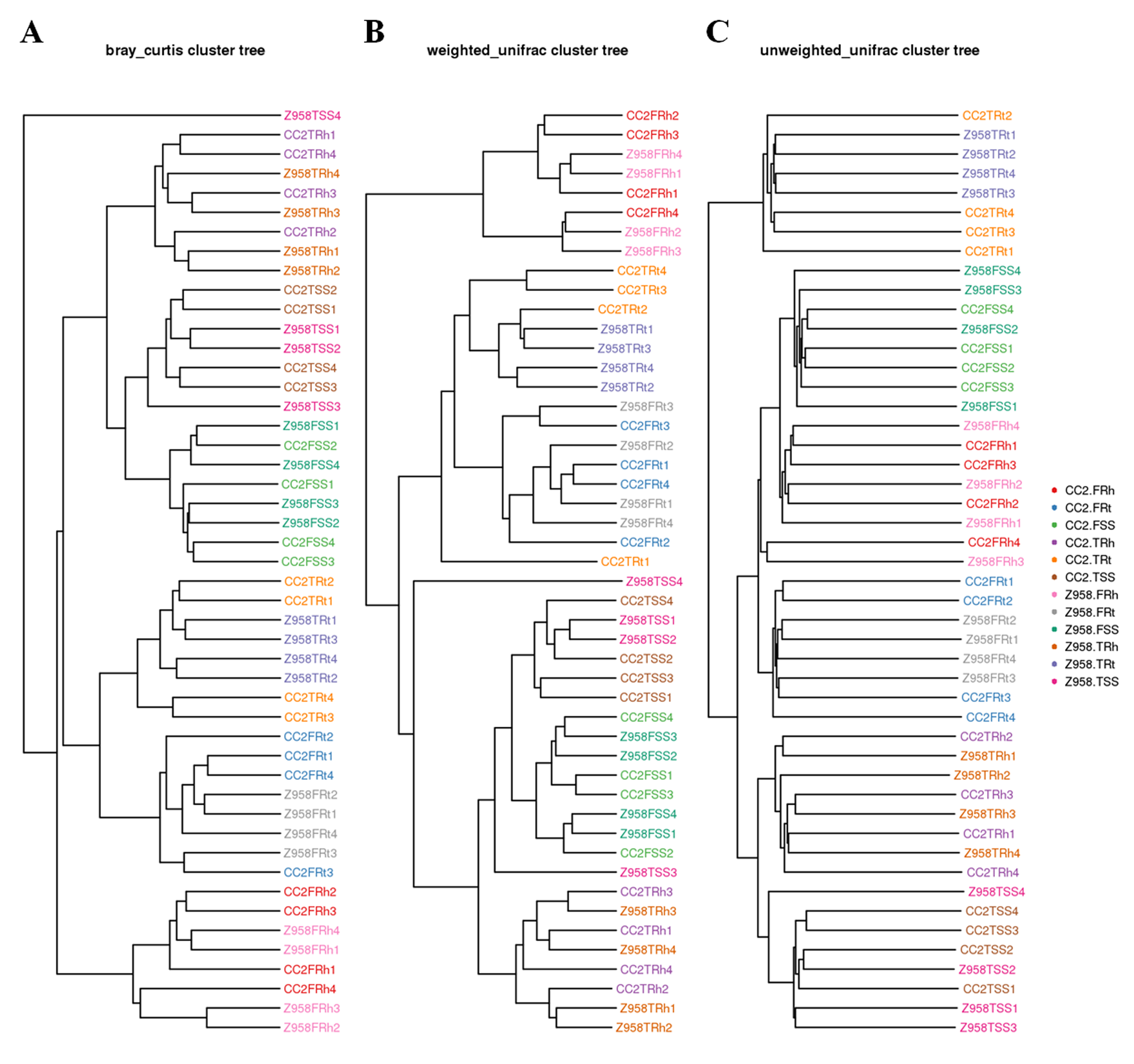

Supplement: Supplementary file 8 [file Image_8.TIF]

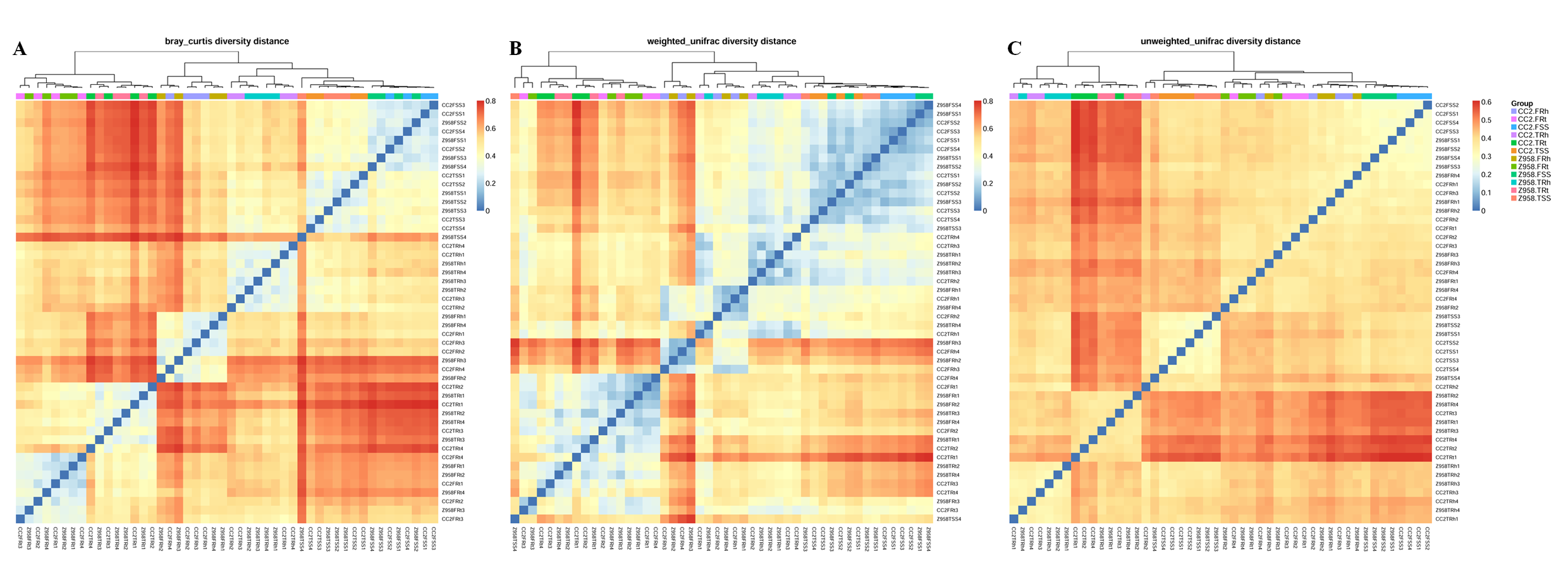

Supplement: Supplementary file 9 [file Image_9.TIF]

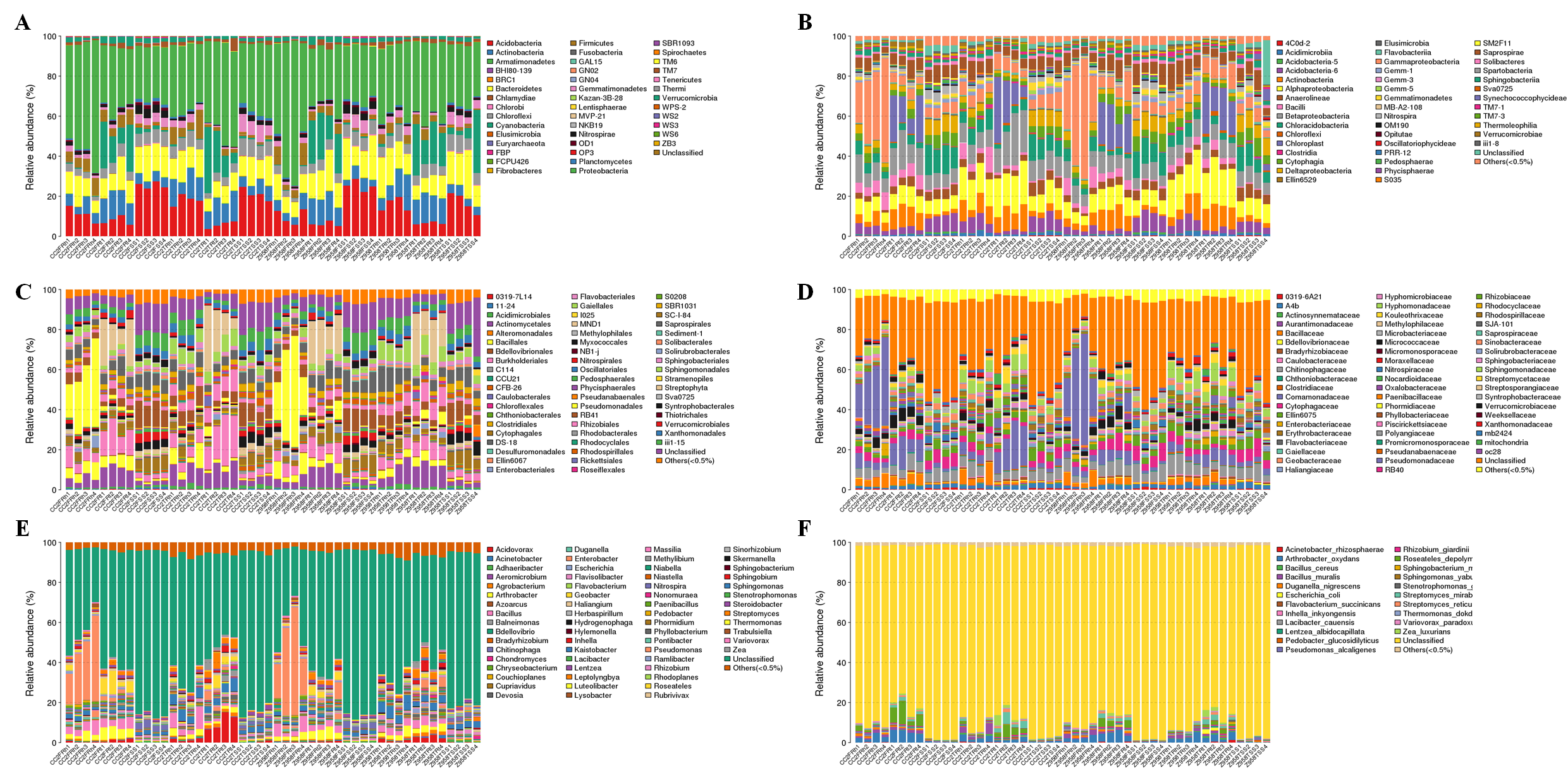

Supplement: Supplementary file 10 [file Image_10.TIF]

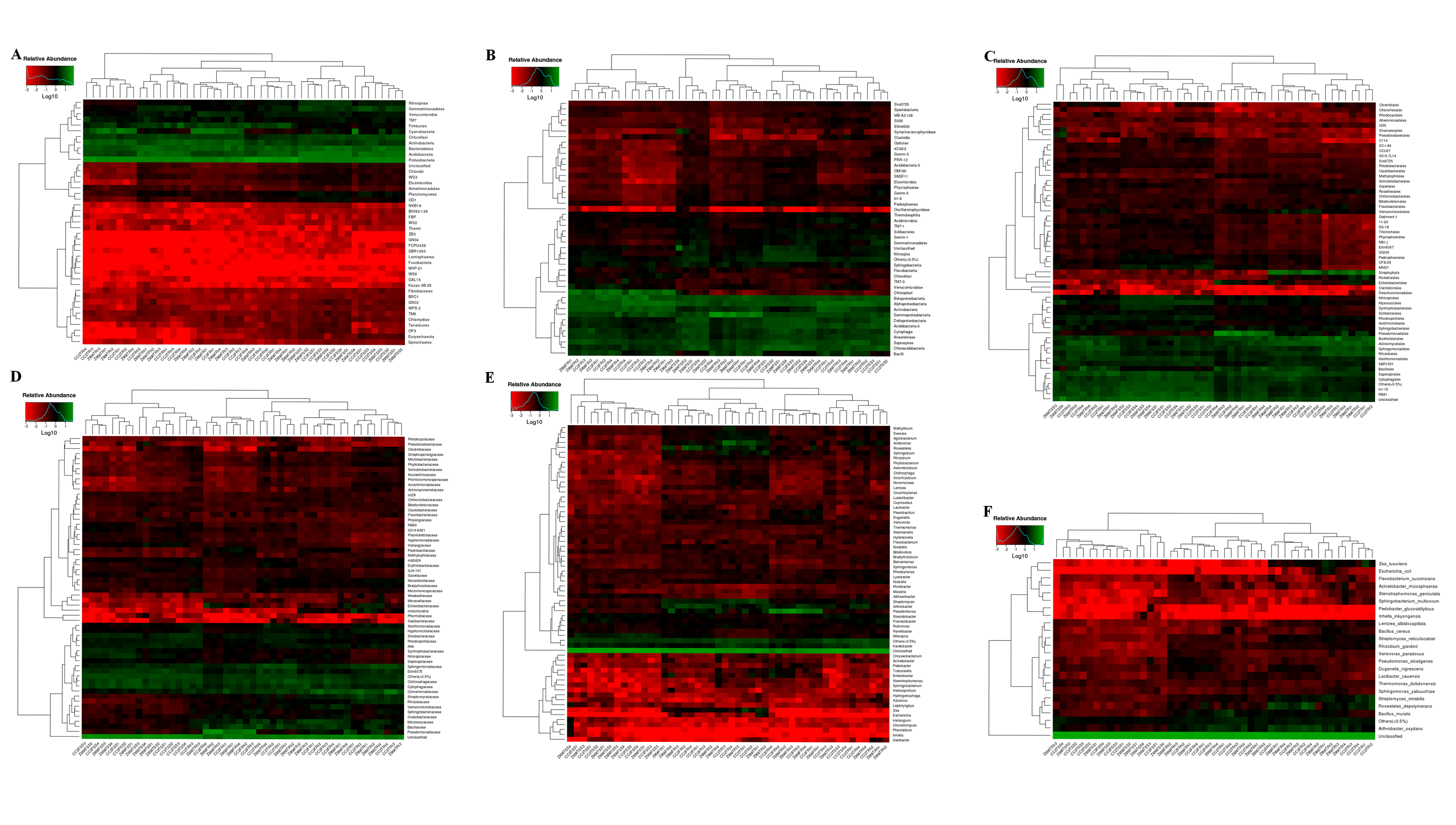

Supplement: Supplementary file 11 [file Image_11.TIF]

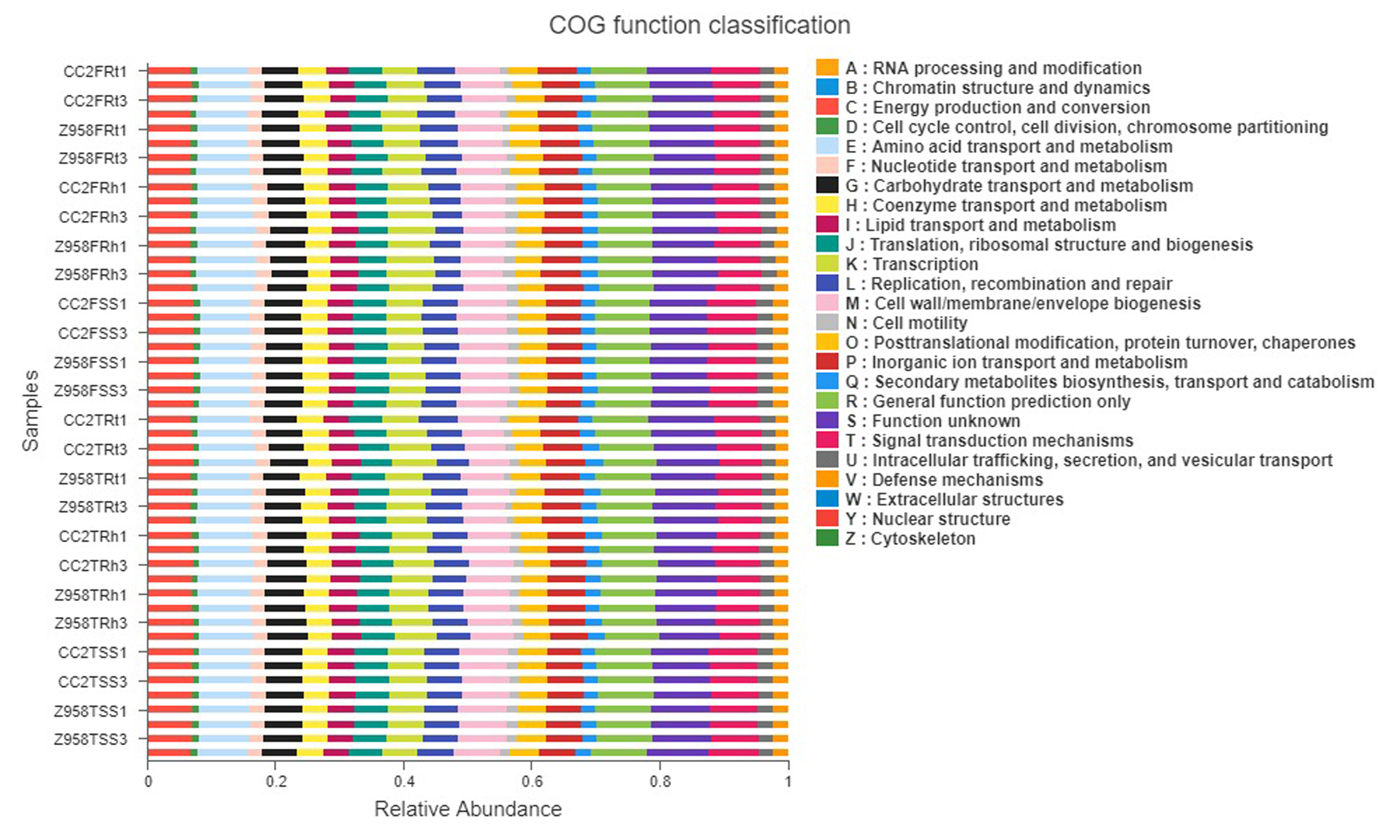

Supplement: Supplementary file 12 [file Image_12.TIF]
